# Structural Modification of Sesquiterpene Lactones via Michael Addition: Improved Bioactivity and Pharmacokinetics

**DOI:** 10.3390/ijms27167374

**Published:** 2026-08-18

**Authors:** Min Woo Ha, Jayun Kang, Sumi Lee, Seung-Mann Paek

**Affiliations:** 1 Jeju Research Institute of Pharmaceutical Sciences, College of Pharmacy, Jeju National University, Jeju 63243, Republic of Korea; minuha@jejunu.ac.kr (M.W.H.);; 2 SynBioChem Convergence Institute, Jeju National University, 102 Jejudaehak-ro, Jeju 63243, Republic of Korea; 3 School of Pharmacy and Institute of New Drug Development, Jeonbuk National University, Jeonju 54907, Republic of Korea; 4 College of Pharmacy and Research Institute of Pharmaceutical Sciences, Gyeongsang National University, Jinju 52828, Republic of Korea

**Keywords:** sesquiterpene lactones, Michael addition, structural optimization, pharmacokinetics, drug-target interactions, α-methylene-γ-lactone

## Abstract

Sesquiterpene lactones (SLs) are privileged medicinal scaffolds, offering potent bioactivities driven by their reactive α-methylene-γ-lactone motif. This review highlights the literature published up to the first half of 2026, detailing the synthetic methodologies utilized to optimize SLs via Michael addition. We critically evaluate how these structural transformations translate into biological and pharmacokinetic improvements. Specifically, the resulting Michael adducts achieve significantly enhanced solubility in aqueous media and highly refined drug–target interactions. Addressing a clear literature gap unaddressed since earlier foundational reviews, this focus review highlights the literature published up to the first half of 2026. By explicitly correlating synthetic strategies with pharmacological outcomes, such as enhanced efficacy and target specificity, this review provides medicinal chemists with a robust framework to accelerate the development of next-generation SL-based therapeutics.

## 1. Introduction

**Figure 1:**
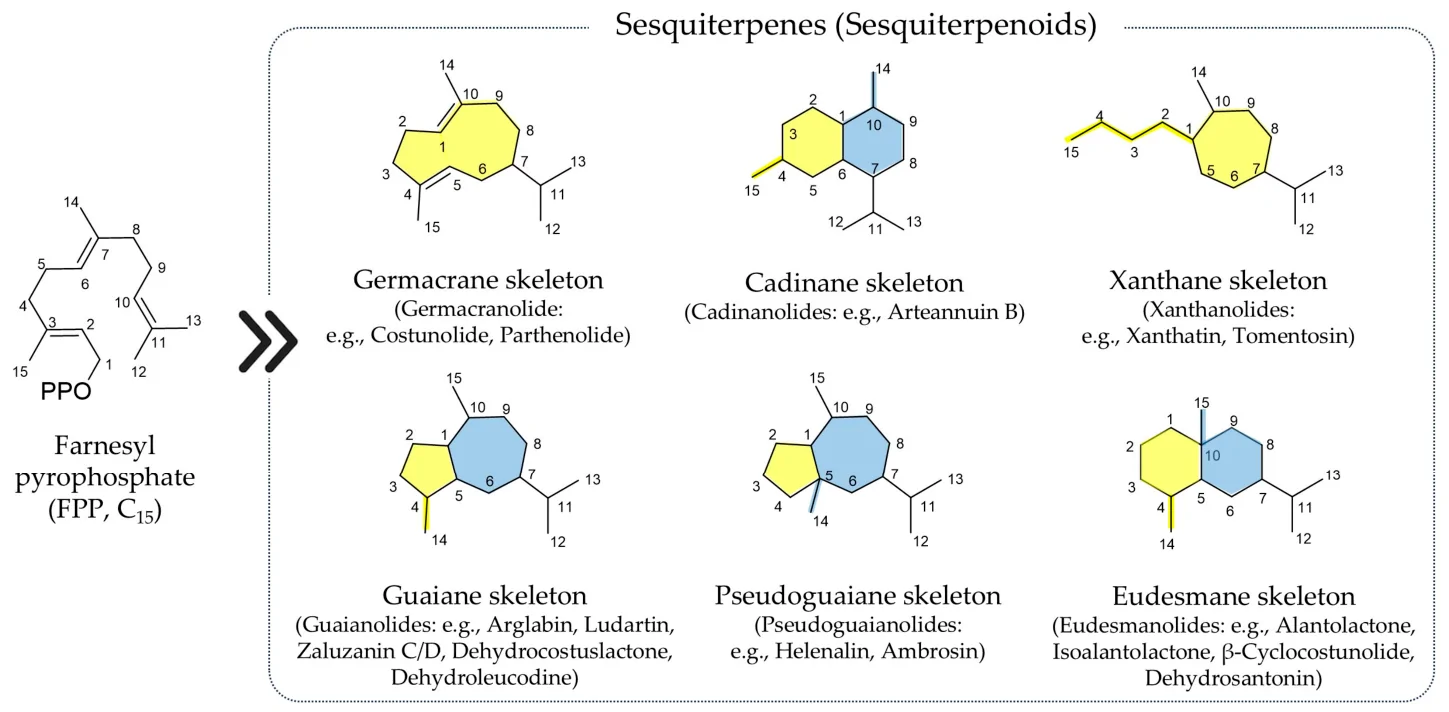
Representative sesquiterpene skeletons and their lactone derivatives.

Sesquiterpene lactones (SLs) are a highly prominent class of plant-derived secondary metabolites that have drawn intense attention from chemists and pharmacologists due to their exceptional anticancer potential [1]. Biogenetically, these compounds originate from the mevalonate or non-mevalonate pathways, where the C_15_ precursor farnesyl pyrophosphate (FPP) undergoes complex enzymatic cyclization and subsequent oxidation to construct the characteristic lactone-fused ring architecture [2]. Structurally, SLs are 15-carbon terpenoids consisting of three isoprene units fused to a lactone ring, typically carrying an α-methylene-γ-lactone motif where an exocyclic double bond is conjugated with a carbonyl function [3]. While more than 100 distinct sesquiterpene skeletal configurations exist in nature, the major and representative structural scaffolds explicitly discussed in this review—such as germacranolides, cadinanolides, xanthanolides, guaianolides, pseudoguaianolides, and eudesmanolides—are summarized and illustrated in Figure 1. Structurally, the exocyclic α-methylene-γ-lactone motif possesses a locked *s*-(*E*) conformation that prevents negative hyperconjugation typical of open-chain acrylates, thereby retaining high electrophilic reactivity. Evaluated by Mayr electrophilicity parameters (*E*), this cyclic framework exhibits significantly enhanced electrophilicity compared to acyclic counterparts. This distinct reactivity window dictates the kinetic balance for reversible Michael additions, enabling efficient covalent targeting of biological thiols and amines while mitigating non-specific proteomic quenches [4].

Through this covalent modification, SLs disrupt intracellular redox equilibrium and induce severe oxidative stress. This action leads to acute thiol depletion and reactive oxygen species (ROS) accumulation, systematically driving malignant cells toward apoptosis. Concurrently, this alkylation downregulates oncogenic signaling by blocking the DNA-binding capability of nuclear factor-kappa B (NF-κB) [5].

Despite these advantages, native SL scaffolds suffer from a narrow therapeutic window, high systemic toxicity, and poor solubility in aqueous media in vivo. To address these limitations, medicinal chemists have utilized a reversible Michael addition to construct aminolactone assemblies. While widely expected to act as water-soluble prodrugs that slowly liberate the parent core in vivo, these amine adducts also serve as distinct, chemically improved derivatives. The introduction of a basic nitrogen tail substantially enhances solubility in aqueous media, refines drug–target interactions, and can impart independent pharmacological mechanisms, presenting an optimized therapeutic entity [6].

This review systematically tracks the relevant literature published up to the first half of 2026, mapping out the synthetic transformations used to optimize SLs via Michael addition. We carefully examine how these structural modifications successfully translate into enhanced solubility in aqueous media, selective bioactivity, and refined drug–target interactions. By explicitly correlating specific synthetic assembly strategies with their precise pharmacological outcomes, this work aims to highlight the clinical and medicinal importance of these optimized scaffolds. To address a clear literature gap that has remained unaddressed since earlier foundational reviews, this focus review systematically tracks the relevant literature up to the first half of 2026, building seamlessly upon earlier foundational SAR surveys [7].

## 2. Michael Adducts of Sesquiterpene Lactones

To overcome the poor solubility in aqueous media and narrow therapeutic windows of native sesquiterpene lactones, structural diversification via a reversible Michael addition has been widely pursued. This approach regioselectively introduces amine or thiol nucleophiles onto the electron-deficient exocyclic β-carbon of the α-methylene-γ-lactone warhead. Table 1 details 14 representative compounds optimized through this strategy, each representing either the earliest synthesized derivatives or pioneering chemical attempts that significantly enhanced pharmacokinetic profiles and expanded therapeutic windows. Highlighting the broad applicability of this strategy, recent studies have further extended this conjugate addition platform to other natural architectures, including pulchellin and grossheimin derivatives [8,9].

### 2.1. Helenalin Derivatives

Helenalin is a pseudoguaianolide sesquiterpene lactone isolated from *Arnica montana* and *Helenium* species. Structurally, it is a bifunctional 15-carbon terpenoid featuring a core α-methylene-γ-lactone motif and an endocyclic α, β unsaturated ketone ring. Although helenalin exhibits potent anti-inflammatory and antitumor activities via biological nucleophile alkylation, its high systemic toxicity and poor solubility in aqueous media have limited clinical development. To address these limitations, a synthetic approach via reversible Michael addition was developed in 1972 [10]. Reacting helenalin with excess dimethylamine in ethanol at 5 °C yielded a water-soluble prodrug adduct that transiently masks the reactive exocyclic double bond.

In vitro evaluation against leukemia and larynx carcinoma cell lines showed an approximate 8-fold reduction in antiproliferative potency for the adduct (ED_50_: 0.083 μg/mL to 0.604 μg/mL) [10]. Despite this lower in vitro potency, the adduct maintained comparable in vivo antitumor efficacy in murine models, showing a survival duration of 13.0 days versus 13.4 days for helenalin [24]. This sustained in vivo efficacy indicates that the reduced target affinity in vitro was successfully compensated for by an improved pharmacokinetic profile.

**Figure 2:**
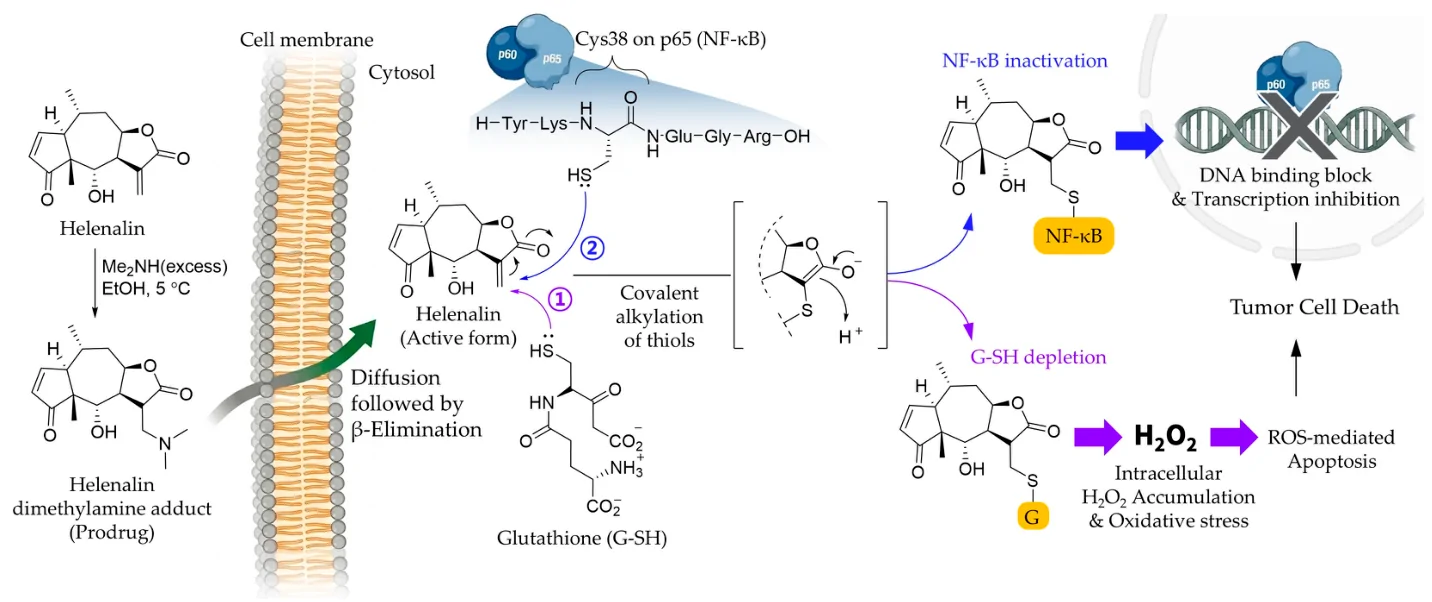
Proposed mechanism of helenalin-induced tumor cell death via dual targeting of intracellular thiol pools: ① scavenging of antioxidant thiols and ② inhibition of transcriptional regulators.

The adduct diffuses across the cell membrane into the cytosol and undergoes spontaneous retro-Michael β-elimination to regenerate the active helenalin core [25] (Figure 2). Once liberated, helenalin targets intracellular thiol pools through a dual mechanism. First, the exocyclic methylene group reacts with intracellular mercaptyl groups, depleting reduced glutathione (G-SH). This disruption impairs the cellular antioxidant defense, causing ROS accumulation and triggering mitochondrial-dependent apoptosis. Second, helenalin covalently alkylates the Cys38 residue on the p65 subunit of NF-κB. This modification blocks the DNA-binding capability of NF-κB and inhibits downstream transcription, cooperatively leading to tumor cell death [26].

### 2.2. Ambrosin Derivatives

Ambrosin is a pseudoguaianolide sesquiterpene lactone that shares the core 5/7-fused bicyclic skeleton with helenalin but lacks the C4-hydroxyl group, altering its molecular geometry and lipophilicity. To improve its low solubility in aqueous media, a bis-amine prodrug strategy was developed in 1995 [11]. Ambrosin was reacted with two equivalents of either pyrrolidine or piperidine in THF at 5 °C for 20 h, followed by conversion to hydrochloride salts. This modification successfully enhanced the solubility in aqueous media of the scaffold.

In screenings across 51 cancer cell lines, both adducts maintained antiproliferative potency comparable to parent ambrosin, with the bis-piperidine adduct showing slightly superior efficacy and chemical stability. This retention of cytotoxicity demonstrates that the bis-amine configuration efficiently delivers the active compound without the typical in vitro potency loss seen in mono-amine adducts.

**Figure 3:**
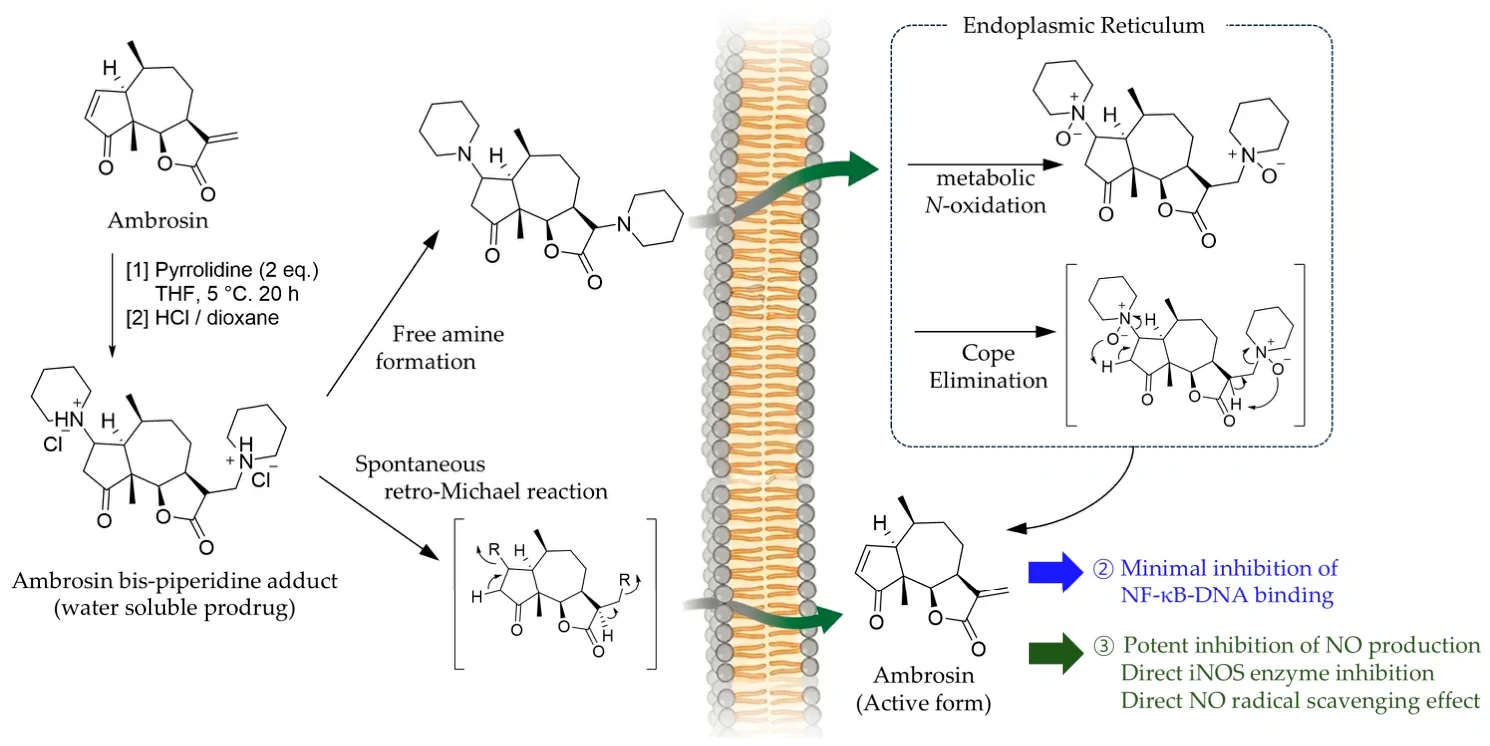
Intracellular activation of ambrosin-prodrug and downstream regulation: ③ potent NO inhibition rather than ② NF-κB–DNA binding suppression.

As shown in Figure 3, the water-soluble adducts diffuse into cells, form free amines, and undergo metabolic *N*-oxidation and Cope elimination within the endoplasmic reticulum to regenerate active ambrosin. Spontaneous retro-Michael reactions also contribute to unmasking the electrophilic core. Once bioactivated, ambrosin exhibits a distinct mechanism from helenalin; it shows minimal inhibition of NF-κB -DNA binding. Instead, it suppresses macrophage-mediated inflammation and proliferation through direct inducible nitric oxide synthase (iNOS) enzyme inhibition and a direct NO radical scavenging effect, significantly decreasing NO production [27].

### 2.3. Costunolide Derivatives

The clinical translation of costunolide is limited by high toxicity, a narrow therapeutic window, and extreme hydrophobicity. To optimize its pharmacokinetic profile and safety index, structural modifications via Michael-type addition have been pursued, as exemplified by the isolation of saussureamine A, a natural L-proline adduct that significantly enhances solubility in aqueous media and provides a chemical rationale for traditional water-based formulations [28]. Absorbed saussureamine A acts as a reversible prodrug, undergoing retro-Michael elimination to liberate the active costunolide core at target physiological sites. Mechanistically, neither compound directly inhibits iNOS enzymatic activity; instead, their interaction with target protein sulfhydryl (-SH) groups leads to a concentration-dependent induction of heat shock protein 72 (HSP 72). Elevated HSP 72 subsequently downregulates the activation and nuclear translocation of NF-κB, suppressing downstream iNOS expression and subsequent NO overproduction [29,30].

**Figure 4:**
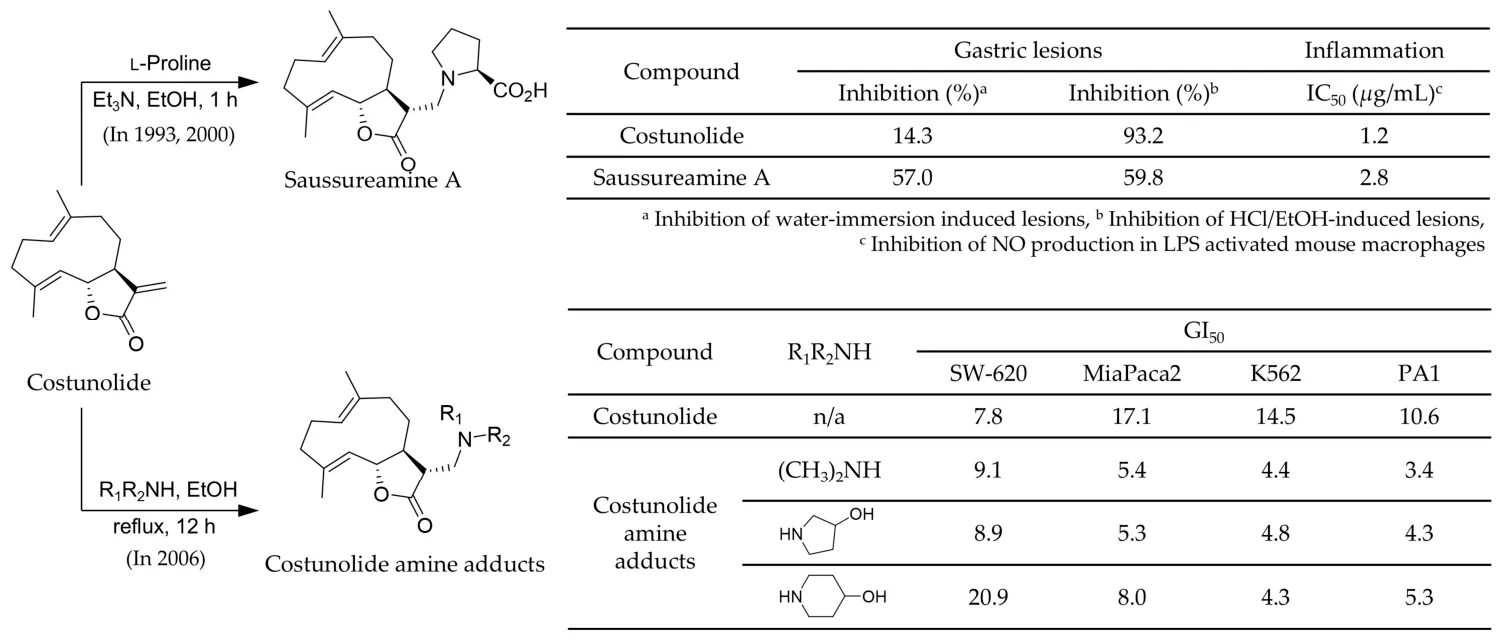
Scheme for the preparation and evaluation of costunolide-amine adducts.

Further optimization of this sesquiterpene scaffold has been achieved through synthetic 13-amino derivatives [12] (Figure 4). Introducing a polar, hydrogen-bonding hydroxyl (OH) tail into cyclic amine rings—as seen in the 4-hydroxypiperidin-1-yl and 3-hydroxypyrrolidin-1-yl adducts—mimics the electronic advantages of a carboxylic acid moiety, refining drug–target interactions and improving cancer cell selectivity over normal fibroblasts. Pharmacologically, the aliphatic *N*, *N*-dimethyl derivative displays broad-spectrum antiproliferative potency against pancreatic, leukemia, and ovarian carcinoma cell lines, along with a significantly improved safety index. Concurrently, the 3-methylpiperidine derivative provides tight tissue-specific selectivity, enhancing cytotoxicity against colon cancer cells with a refined safety index exceeding 2. These modifications effectively expand the therapeutic window, offering a robust approach for designing optimized derivatives and water-soluble prodrugs.

### 2.4. Alantolactone Derivatives

The structural modification of alantolactone and isoalantolactone via the introduction of a diethylamine moiety represents a strategic approach to enhancing both pharmacological potency and PK profiles. Natural sesquiterpene lactones frequently suffer from premature, non-specific reactions with endogenous nucleophiles prior to reaching their therapeutic targets, leading to poor bioavailability. By converting these highly reactive parent lactones into their respective diethylamine adducts, this structural alteration improves hydrophilicity and basicity, providing a chemical scaffold optimized for enhanced solubility and systemic circulation. Notably, the diethylamine adduct of alantolactone completely preserves the potent cytotoxicity (IC_50_ = 0.7 μM) against K562 human leukaemia cells, while the isoalantolactone derivative maintains a low-micromolar level of activity (IC_50_ = 2 μM), demonstrating that the spatial bulkiness at the C-11 position does not restrict pharmacological efficacy [13].

**Figure 5:**
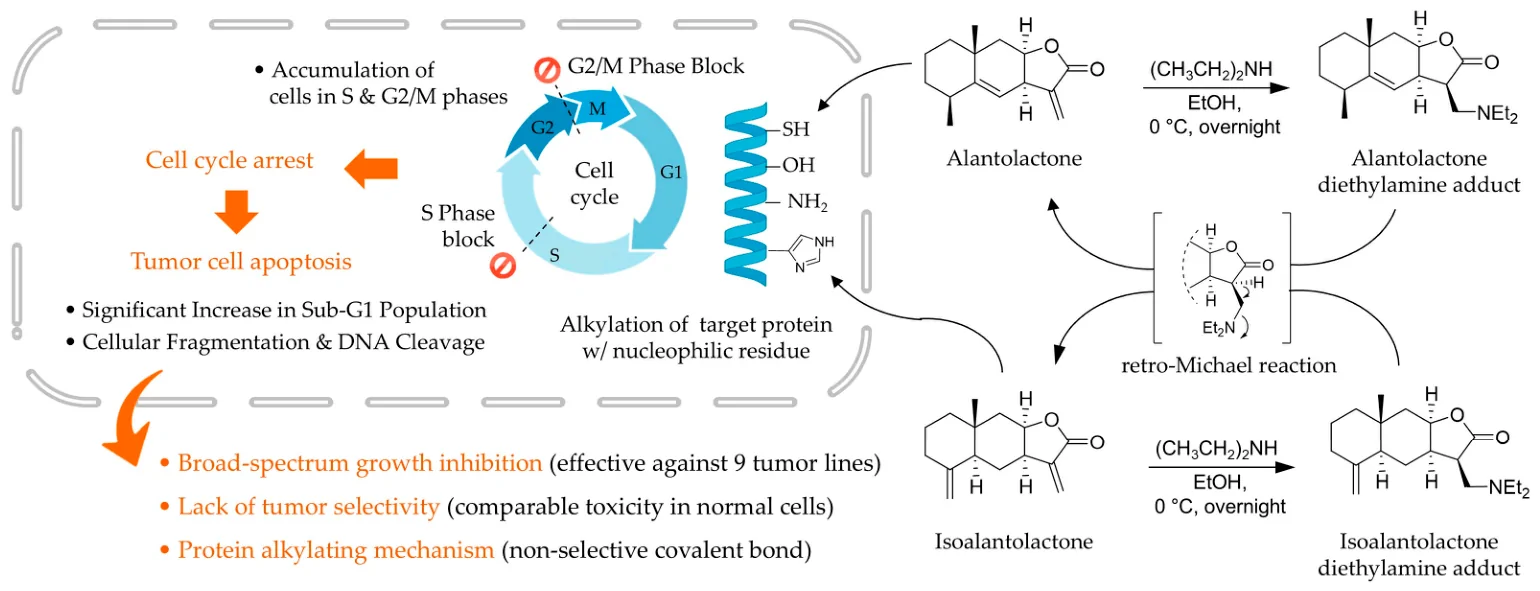
Cytotoxic mechanism and cell cycle regulation of alantolactone derivatives.

The experimental cytotoxicity profiles showing that the diethylamine derivatives retain a powerful potency nearly identical to that of their parent counterparts strongly validate the occurrence of an in vivo reversible Michael-type reaction (Figure 5). This comparable bioactivity proves that the amine alteration at the C-11 position acts as a temporary masking group rather than compromising the pharmacophore, undergoing intracellular retro-Michael elimination to successfully regenerate the active parent lactones. These liberated electrophilic cores subsequently attack target proteins to form non-specific covalent bonds with nucleophilic residues—predominantly cysteine thiol groups, alongside hydroxyl and nitrogenous side chains. This widespread proteomic and DNA alkylation disrupts essential biological machinery, triggering robust S and G2/M phase cell cycle arrest and inducing apoptosis, as evidenced by a sharp elevation in the sub-G1 population.

Building upon these structural optimization strategies, a subsequent study in 2019 expanded the chemical space of alantolactone by synthesizing a novel series of semi-synthetic thiol derivatives via regioselective Michael addition to explore enhanced anti-inflammatory potentials [31]. This pharmacological screening demonstrated that these sulfur-appended architectures significantly attenuate lipopolysaccharide (LPS)-induced pro-inflammatory mediators—including nitric oxide (NO), interleukin-6 (IL-6), and tumor necrosis factor-α (TNF-α)—in both in vitro macrophage models and in vivo murine sepsis models without altering baseline cellular viability. Aligning with these structural optimization efforts, conjugating substituted piperazines to natural alantolactones was recently shown to afford amino derivatives with markedly enhanced water solubility and antiproliferative profiles [32].

### 2.5. Parthenolide Derivatives

**Figure 6:**
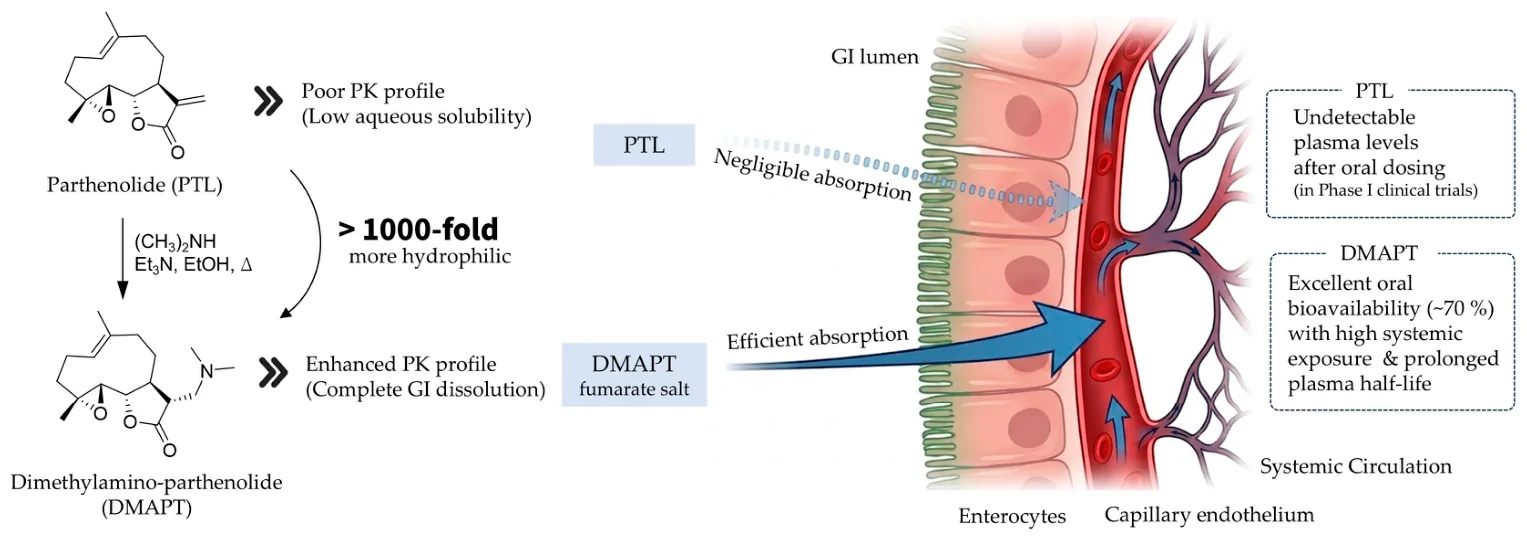
Chemical modification and comparative oral pharmacokinetics of parthenolide and DMAPT.

Among various SLs, parthenolide (PTL) is arguably the most extensively studied archetype. To overcome the critical pharmacokinetic barriers inherent to this native scaffold, chemical modifications have been strategically employed. Native PTL features an electrophilic α-methylene-γ-lactone core that functions as a Michael acceptor; however, its clinical development has been severely bottlenecked by high non-specific plasma protein binding and poor solubility in aqueous media, which lead to hydrophobic aggregation and negligible absorption within the gastrointestinal (GI) lumen. Indeed, Phase I clinical trials confirmed that native PTL remains completely undetectable in patient plasma samples following oral dosing. To address these delivery limitations, the chemical introduction of a dimethylamine group was pioneered, generating the water-soluble amine adduct dimethylamino-parthenolide (DMAPT) as a fumarate salt platform [14]. This targeted dimethylamine addition achieves a greater than 1000-fold increase in hydrophilicity relative to the parent compound, ensuring complete dissolution in the GI tract, successful permeation through the enterocyte brush border and capillary endothelium, and a remarkable oral bioavailability of approximately 70% with high systemic exposure and a prolonged plasma half-life across in vivo models [33] (Figure 6). Expanding upon these amine-based approaches, stereodivergent double Thia-Michael additions on related sesquiterpene architectures were recently reported, demonstrating that thia-conjugation can effectively decouple non-specific cytotoxicity from desired anti-inflammatory efficacy [34].

**Figure 7:**
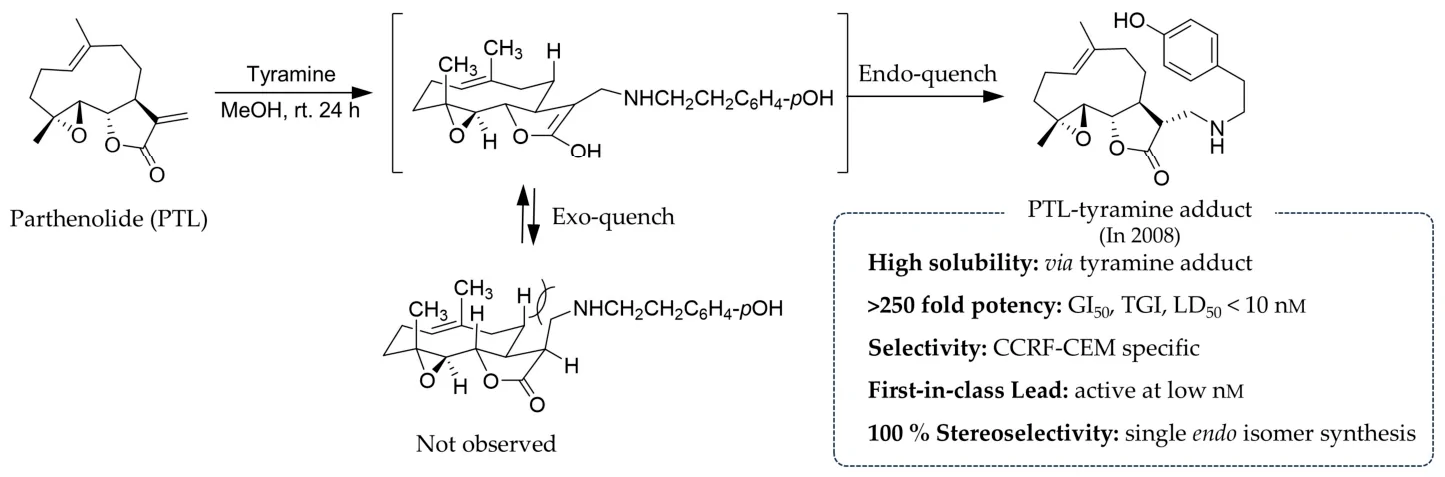
Stereoselective synthesis of the PTL-tyramine adduct: enhanced solubility and discovery of a first-in-class lead with nanomolar antileukemic activity.

While earlier modifications of parthenolide focused on creating water-soluble prodrugs, specific Michael adducts can inherently act as potent therapeutic leads. Synthetically, functionalizing the α-methylene-γ-lactone moiety via amine conjugate addition offers remarkable precision. A 2008 study demonstrated that introducing tyramine to parthenolide generates a privileged scaffold with complete diastereoselectivity [35] (Figure 7). Driven by thermodynamic control, the reaction exclusively yields the endo-quenched product. The alternative exo-quenched isomer is not observed, as it is energetically disfavored by severe 1,3-diaxial steric strain. This synthetic elegance ensures absolute stereocontrol, avoiding unwanted isomeric mixtures and consistently producing the stable (11*R*)-diastereomer.

Pharmacological evaluation revealed that this structural alteration directly amplifies intrinsic bioactivity rather than merely improving pharmacokinetics. When screened against 60 human cancer cell lines, the tyramine adduct exhibited exceptional antileukemic properties. It acted as a potent cytostatic and cytotoxic agent against human acute lymphoblastic leukemia (CCRF-CEM) cells at concentrations below 10 nM. This nanomolar efficacy sharply contrasts with typical parthenolide analogs, which generally require micromolar concentrations to induce cell death. Furthermore, this toxicity was highly specific to CCRF-CEM cells, showing significantly reduced effectiveness against the remaining 59 cell lines. These findings highlight the parthenolide–tyramine adduct as a highly privileged structure, merging synthetic elegance with robust target selectivity and potency.

**Figure 8:**
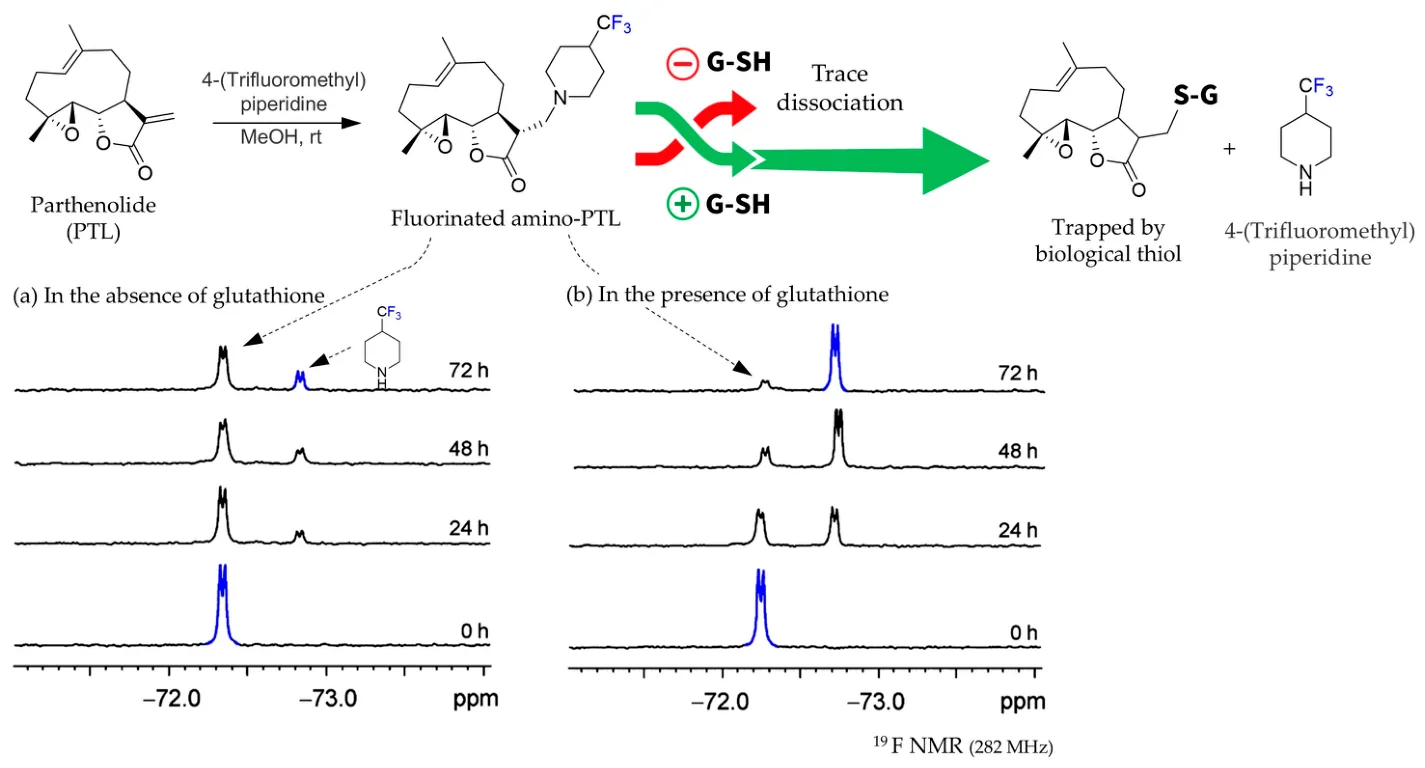
Time-course ^19^F NMR analysis of glutathione-dependent retro-Michael reaction. ^19^F NMR spectra monitoring the release of 4-(trifluoromethyl)piperidine from the fluorinated amino-PTL over 72 h at 80 °C (pH 7.4): (**a**) in the absence of glutathione and (**b**) in the presence of 13.5 mM glutathione.

To further elucidate the intracellular activation mechanism of aminoparthenolide derivatives, a fluorinated analogue strategically designed as a ^19^F NMR probe was introduced in 2011 [36] (Figure 8). By conjugating parthenolide with 4-(trifluoromethyl)piperidine via Michael addition, a derivative was synthesized that successfully maintained potent antileukemic activity comparable to the parent natural product. The incorporation of the trifluoromethyl group enabled the direct, quantitative observation of the retro-Michael reaction using a highly sensitive, deuterium-free ^19^F NMR method. This innovative approach allowed for the precise monitoring of the fluorinated amine’s release from the parthenolide scaffold under biologically relevant conditions without requiring internal standards or deuterated solvents. The time-course ^19^F NMR tracking provided critical mechanistic insights into the prodrug’s behavior. The spectra quantitatively demonstrated that the elimination of the fluorinated amine occurs significantly faster and to a much greater extent in the presence of glutathione compared to its absence. This G-SH-driven accelerated release is governed by the reversible nature of the retro-Michael reaction coupled with competitive nucleophilic trapping. In solution, the amino-PTL exists in a dynamic equilibrium with the dissociated free amine and the regenerated electrophilic parthenolide core. When glutathione is present, its highly nucleophilic thiol group rapidly and preferentially traps the exposed Michael acceptor, outcompeting the readdition of the amine. This irreversible trapping consumes the active core, preventing recombination and effectively driving the chemical equilibrium toward continuous dissociation of the adduct. Consequently, in cancer cells characterized by elevated glutathione levels, this thiol-mediated activation accelerates the unmasking of the reactive Michael acceptor, contributing to the targeted cytotoxicity and cellular selectivity of the prodrug.

Although DMAPT successfully resolved the extreme hydrophobicity of native parthenolide, there remained a critical need to further optimize its overall pharmacokinetic (DMPK) profile and mitigate potential off-target liabilities, such as hERG channel-mediated cardiotoxicity. As part of a dedicated effort to develop effective targeted therapies for chronic lymphocytic leukemia (CLL)—particularly for treatment-refractory cases—medicinal chemists strategically shifted toward incorporating more sophisticated heterocyclic appendages. Building on this rationale, a 2019 study introduced an advanced, heterocycle-modified parthenolide derivative designed to achieve an optimal balance between potent anti-CLL efficacy and in vivo safety [37].

**Figure 9:**
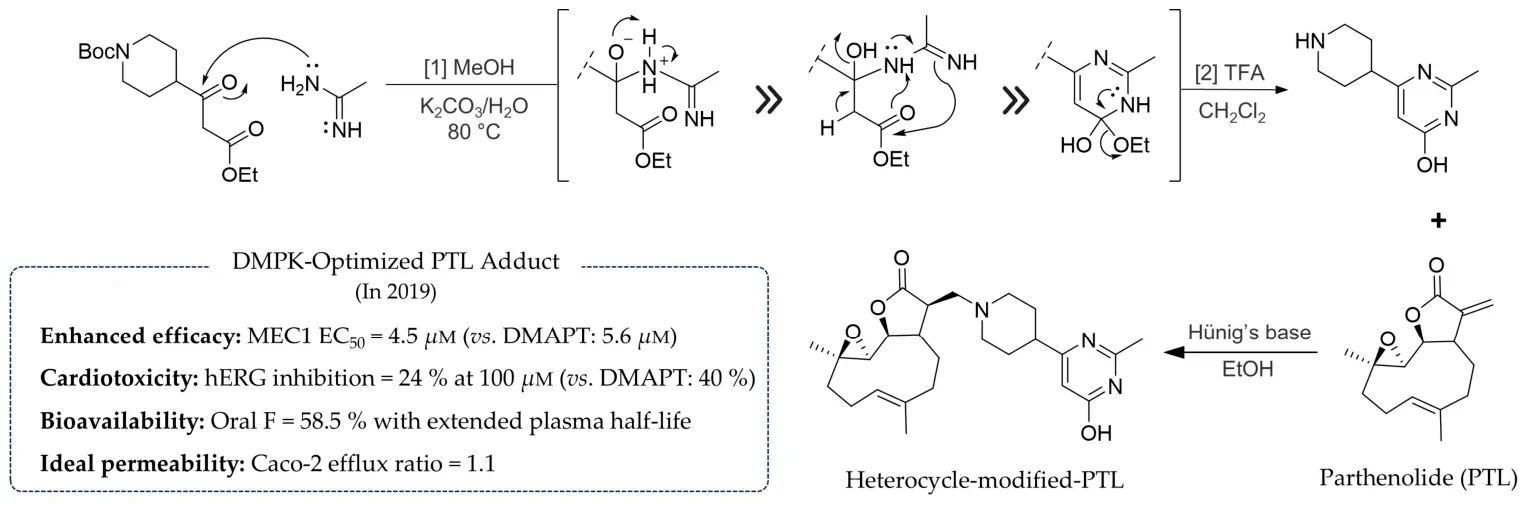
Synthesis and pharmacological profile of the advanced, DMPK-optimized PTL derivative.

The synthesis of this advanced, DMPK-optimized parthenolide adduct begins with the strategic construction of a customized pyrimidine–piperidine amine. An *N*-Boc-protected β-keto ester undergoes a cyclization reaction with acetamidine under basic conditions to form the substituted pyrimidine core. Subsequent Boc deprotection utilizing trifluoroacetic acid (TFA) yields the free secondary amine intermediate. Finally, this customized amine is conjugated to the highly reactive exocyclic α-methylene-γ-lactone of PTL via a regioselective Michael addition in the presence of Hünig’s base, successfully generating the target heterocycle-modified PTL adduct (Figure 9).

This advanced structural modification translates into a highly refined pharmacological profile, effectively overcoming the pharmacokinetic limitations of early-generation derivatives. The optimized adduct retains potent efficacy against CLL, demonstrating an EC_50_ of 4.5 µM against the treatment-refractory MEC1 CLL cell line. More importantly, it achieves an exceptional oral bioavailability of 58.5% with an extended plasma half-life and an ideal Caco-2 efflux ratio of 1.1. Concurrently, the sophisticated heterocycle appendage mitigates cardiotoxicity risks, restricting hERG channel inhibition to merely 24% at 100 µM. Ultimately, this synthetic advancement establishes a highly privileged therapeutic lead that maximizes both systemic exposure and in vivo safety.

Continuing their concerted efforts to combat CLL, the research group further expanded the structural diversity of PTL derivatives in 2020 by synthesizing aniline-containing adducts [38]. Recognizing that *N*-aryl modifications were largely underexplored due to inherent synthetic challenges, they established a novel squaric acid-catalyzed 1,4-conjugate addition methodology to successfully conjugate anilines to the PTL scaffold. Although these aniline derivatives—particularly the phenol-containing variants—exhibited moderate anti-leukemic activity with LC_50_ values ranging from 11.7 to 12.9 µM in MEC1 cells, they did not surpass the potency of the earlier heterocycle-modified lead. However, this study successfully expanded the accessible chemical space of the PTL scaffold. Ultimately, it provided valuable structure–activity relationship data, delineating the boundaries of *N*-aryl conjugations and guiding future rational drug design for CLL-targeted therapies.

### 2.6. α-Santonin Derivatives

**Figure 10:**
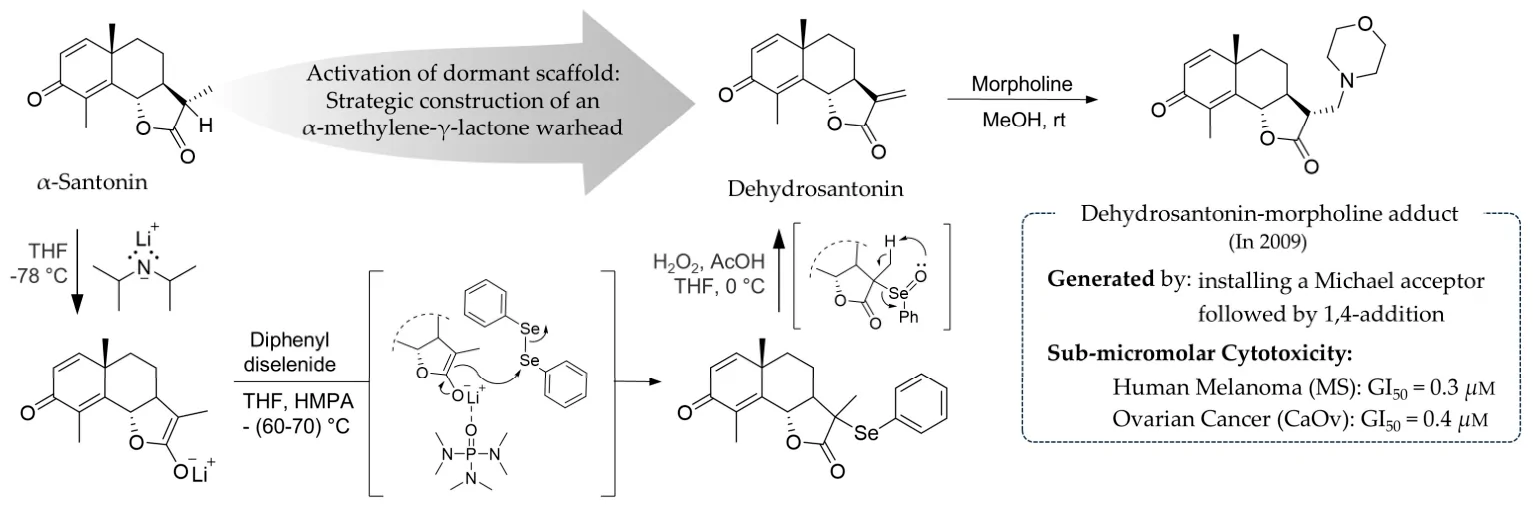
Strategic reactivation of the α-santonin scaffold via α-methylene-γ-lactone installation and 1,4-addition.

Driven by the motivation to develop amine conjugates using the abundant eudesmanolide α-santonin as a core scaffold, a research group in 2009 explored the reactivation of this dormant molecule. Because native α-santonin lacks the requisite α-methylene-γ-lactone warhead, researchers strategically installed an electrophilic exocyclic olefin at the molecular terminus [15]. This was achieved through a two-step chemical sequence: first, a strong base generated a lithium enolate, and the addition of HMPA solvated the lithium cation, maximizing the enolate’s nucleophilicity to react with diphenyldiselenide. The resulting α-phenylseleno intermediate was then oxidized with H_2_O_2_, facilitating selenium elimination to successfully yield dehydrosantonin equipped with a reactive Michael acceptor. With this handle installed, a series of aminosantonin adducts were synthesized via 1,4-aza-Michael addition. While the overall cytotoxicity of the library was moderate, the morpholine adduct exhibited notable sub-micromolar activity (GI_50_ = 0.3~0.4 μM) against human melanoma and ovarian cancer cell lines, successfully demonstrating that biologically dormant natural cores can be chemically awakened through strategic terminal modifications. Complementing these aza-Michael transformations, Michael-type thiol adducts of exocyclic methylene α-santonin analogues have also been successfully developed as potent anticancer leads (Figure 10) [39].

### 2.7. Arglabin Derivatives

**Figure 11:**
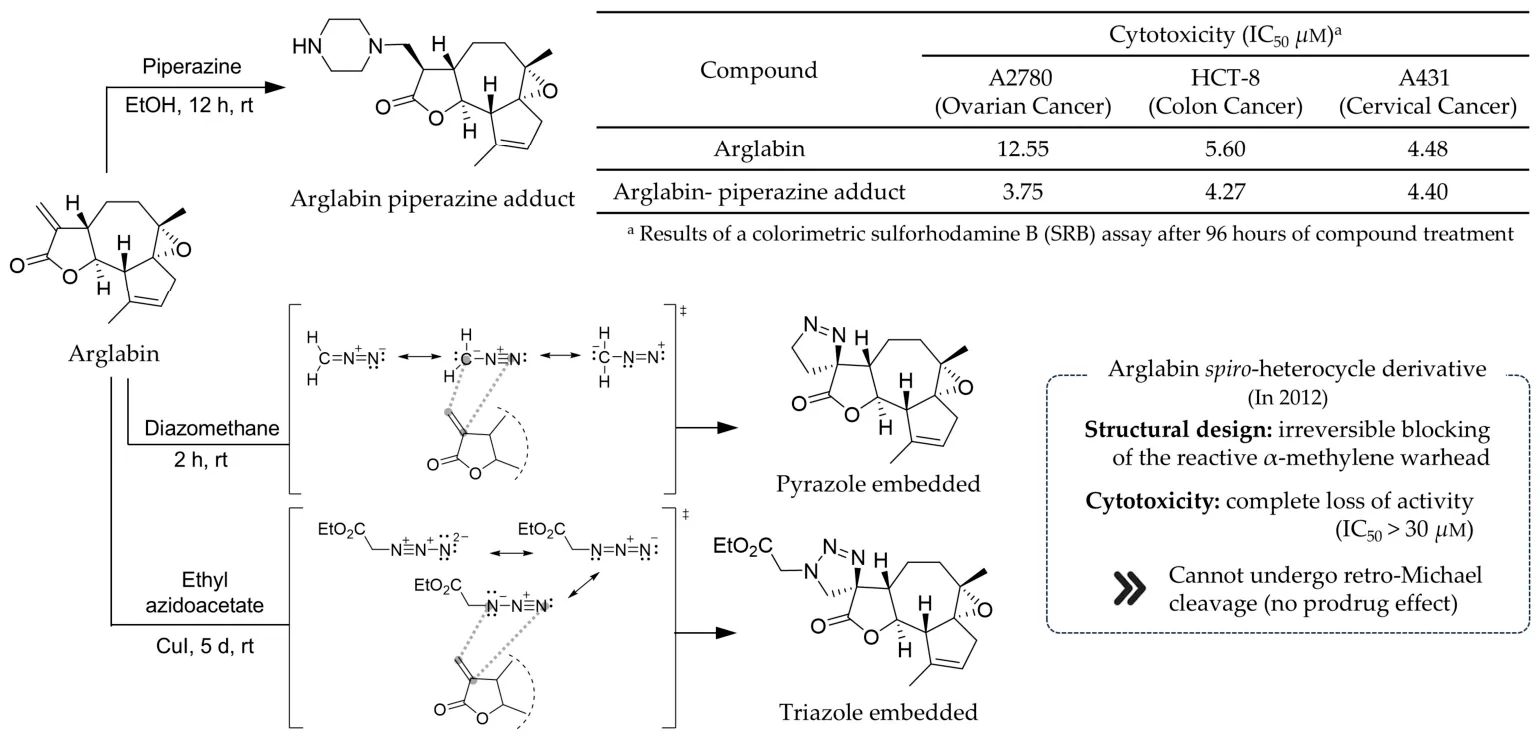
Divergent functionalization of arglabin: enhanced cytotoxicity of the piperazine adduct against ovarian cancer cells versus inactive rigid *spiro*-heterocycle derivatives.

Arglabin derivatives have been strategically developed via regioselective Michael-type additions to act as farnesyl-transferase inhibitors targeting the Ras pathway. For instance, an arglabin piperazine adduct exhibits potent cytotoxic activity and enhanced selectivity, achieving an improved IC_50_ of 3.75 μM against A2780 ovarian cancer cells compared to the parent compound’s 12.55 μM. This piperazine adduct also demonstrates strong cytotoxicity against HCT-8 colon and A431 cervical cancer cell lines. Conversely, structural modifications that irreversibly block the reactive *α*-methylene warhead, such as pyrazole- or triazole-embedded *spiro*-heterocycle derivatives, result in a complete loss of biological activity (IC_50_ > 30 μM). This loss of efficacy highlights the importance of a reversible linkage, as these permanently blocked *spiro*-heterocycles cannot undergo the necessary retro-Michael cleavage to function as active prodrugs [13]. Further advancing the synthetic methodology for this scaffold, phosphine-catalyzed Michael additions were recently introduced to efficiently yield bioactive arglabin adducts under mild organocatalytic conditions (Figure 11) [40].

### 2.8. Ludartin Derivatives

**Figure 12:**
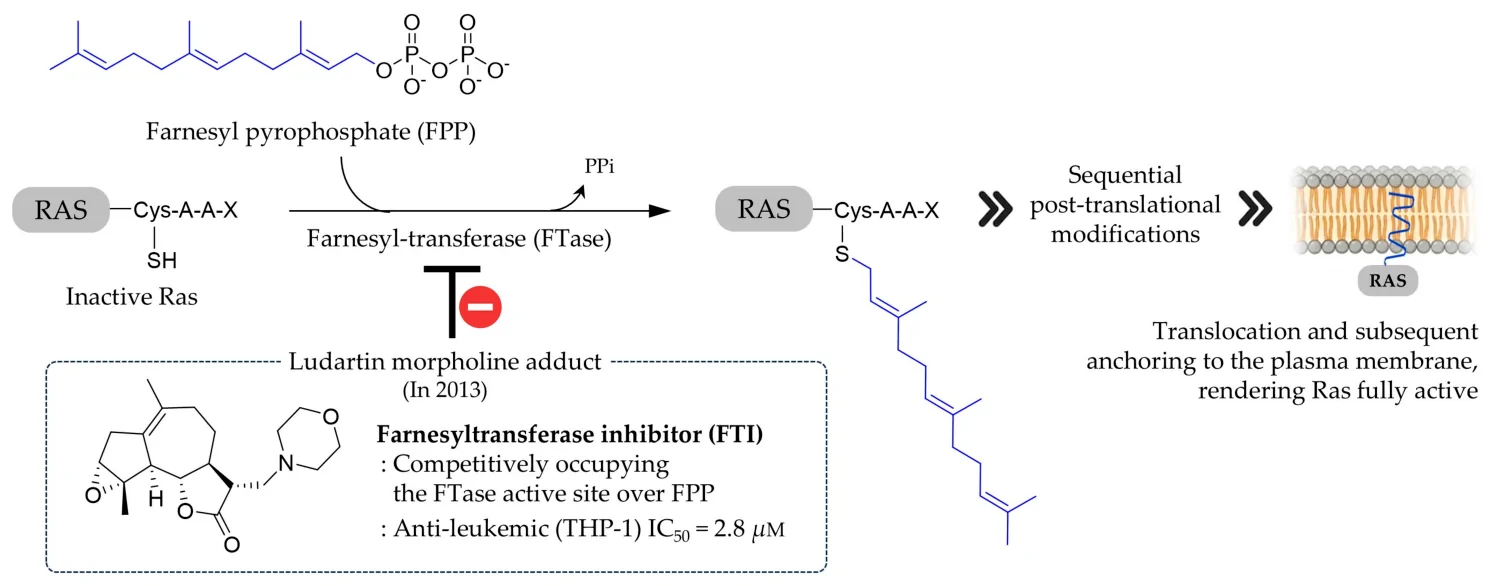
Mechanism of Ras protein farnesylation and its competitive inhibition by the ludartin morpholine adduct.

In 2013, the first series of ludartin amine derivatives was synthesized and confirmed to possess potent anticancer activity [17]. As depicted in Figure 12, the ludartin morpholine adduct functions as a competitive farnesyltransferase inhibitor (FTI) targeting the Ras activation pathway, sharing an identical mechanism of action with arglabin amino derivatives [41]. By competitively occupying the FTase active site over the natural substrate, farnesyl pyrophosphate (FPP), these adducts effectively block the farnesylation of Ras proteins. This targeted inhibition prevents the sequential post-translational modifications essential for Ras translocation and subsequent anchoring to the plasma membrane. Consequently, they successfully prevent the Ras protein from rendering fully active, providing a solid mechanistic basis for their potent anti-tumor efficacies.

### 2.9. Zaluzanin D/C Derivatives

**Figure 13:**
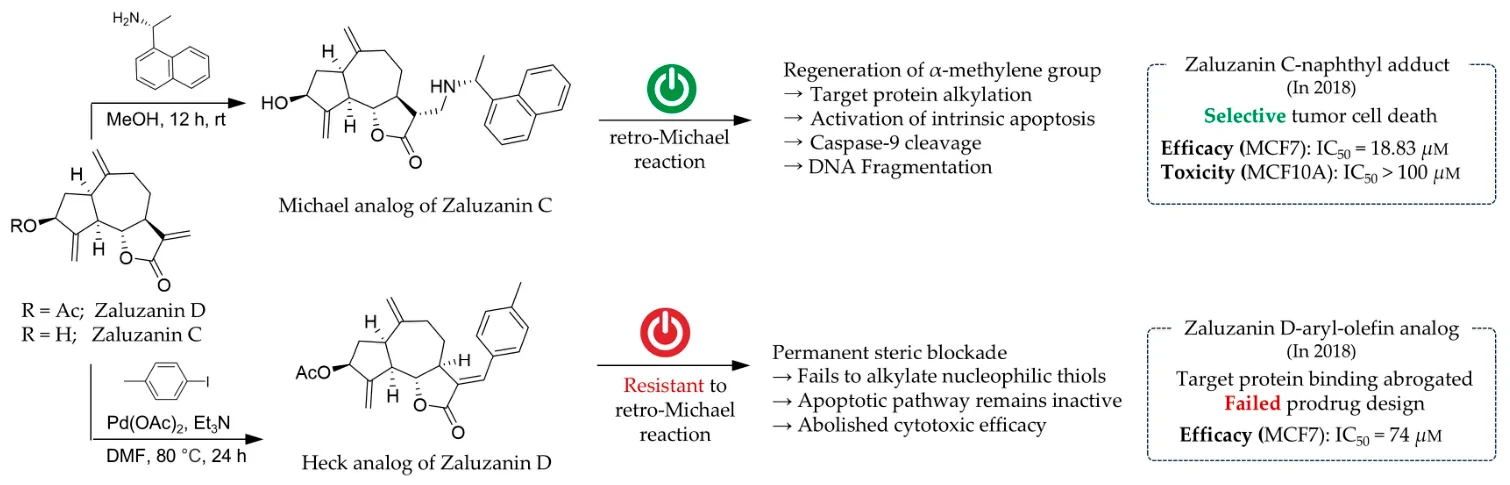
Comparative mechanistic rationale of Zaluzanin derivatives: reversible prodrug activation of the Michael adduct vs. permanent steric blockade in the Heck arylation analog.

Zaluzanin D and its deacetylated counterpart, Zaluzanin C, are guaianolide sesquiterpene lactones isolated from *Vernonia arborea*. To improve their pharmacokinetic properties and mitigate off-target toxicity, a series of aza-Michael adducts was synthesized in 2018 [18]. Among them, the Zaluzanin C-naphthyl adduct emerged as a highly efficient prodrug. This adduct undergoes intracellular retro-Michael cleavage to regenerate the reactive α-methylene-γ-lactone core, which subsequently induces selective tumor cell death via the intrinsic apoptotic pathway while sparing normal epithelial cells. As illustrated in Figure 13, the critical requirement for this reversible masking strategy was validated by comparing these Michael adducts with a series of irreversible Heck arylation analogs. By covalently attaching bulky aryl groups to the exocyclic double bond, such as in the Zaluzanin D-aryl-olefin analog, the resulting permanent steric blockade abrogated target protein alkylation and abolished cytotoxic efficacy. This confirms that the transient, reversible nature of the Michael addition is essential for successful prodrug design.

### 2.10. Dehydrocostuslactone & β-Cyclocostunolide Derivatives

**Figure 14:**
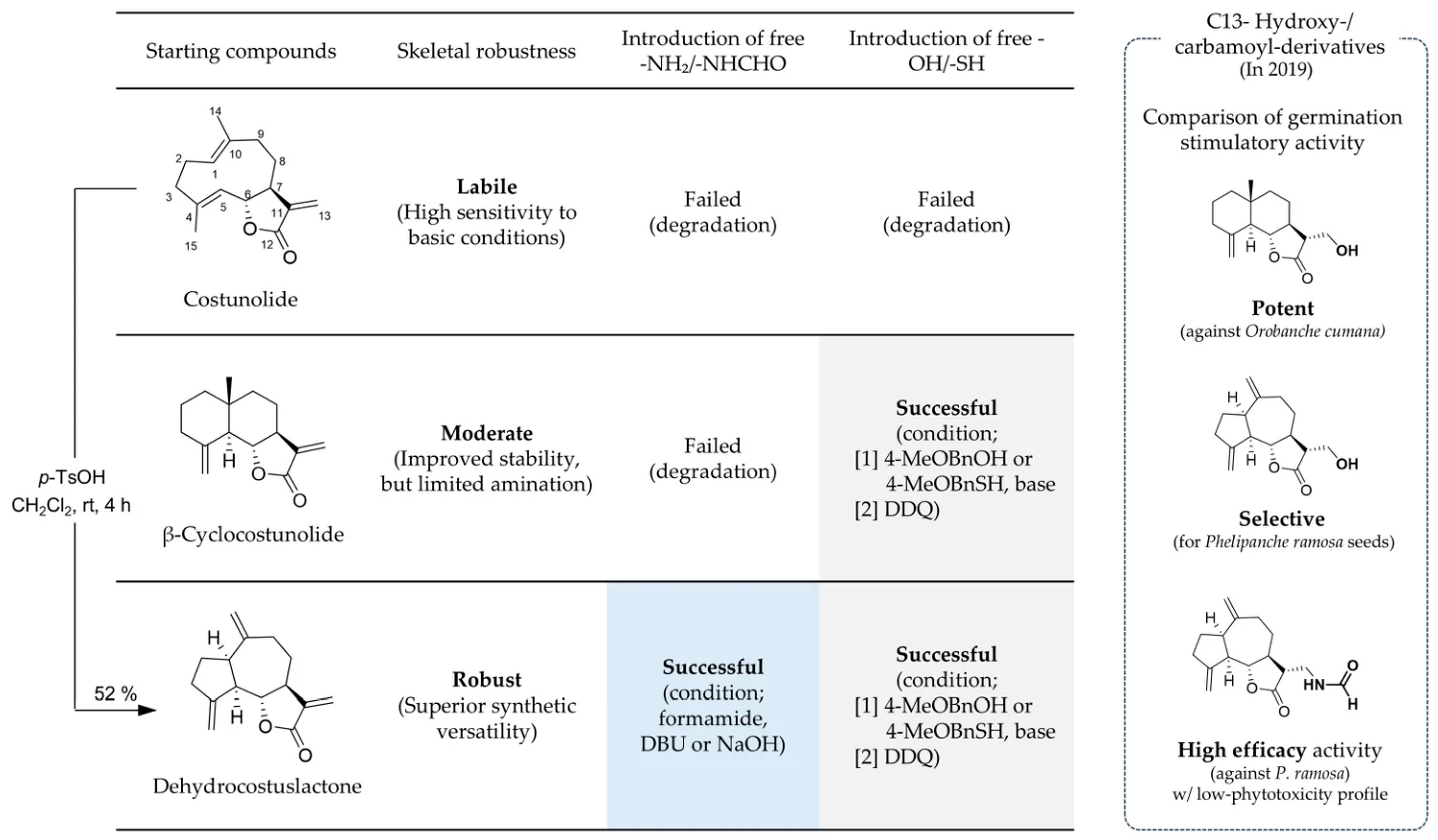
Skeletal robustness, synthetic versatility, and germination stimulatory activity of C-13 functionalized derivatives.

The exploration of the α-methylene-γ-lactone moiety in SLs has long been a focal point for understanding their bioactivity, yet previous synthetic efforts were largely constrained to the introduction of dialkyl amines. Moving beyond these limitations, a recent study demonstrated a robust strategy for the direct installation of free amine, hydroxyl, and thiol functional groups onto the scaffold [19]. The researchers initially targeted costunolide for direct modification at the C-13 position; however, the intrinsic instability of its 10-membered ring system proved prohibitive. To overcome this structural hurdle, they utilized acid-catalyzed conditions to obtain the more stable β-cyclocostunolide, while also employing dehydrocostuslactone as a structurally rigid platform to facilitate the successful incorporation of these free functional groups. As summarized in Figure 14, this approach allowed for the efficient derivatization of the lactone fragment. The resulting library of compounds was systematically evaluated for their bioactivity against parasitic weeds, revealing significant structure–activity relationships. This work represents a notable advancement in the field, as it expands the chemical space of sesquiterpene lactones and demonstrates that a strategic modification of the scaffold can significantly enhance the library’s potential for developing new bioactive agents. Building on these functionalization strategies, ongoing investigation in this domain has recently yielded Thia-Michael adducts of dehydrocostuslactone with potent anticancer activity and validated in silico profiles [42].

### 2.11. Xanthatin Derivatives

**Figure 15:**
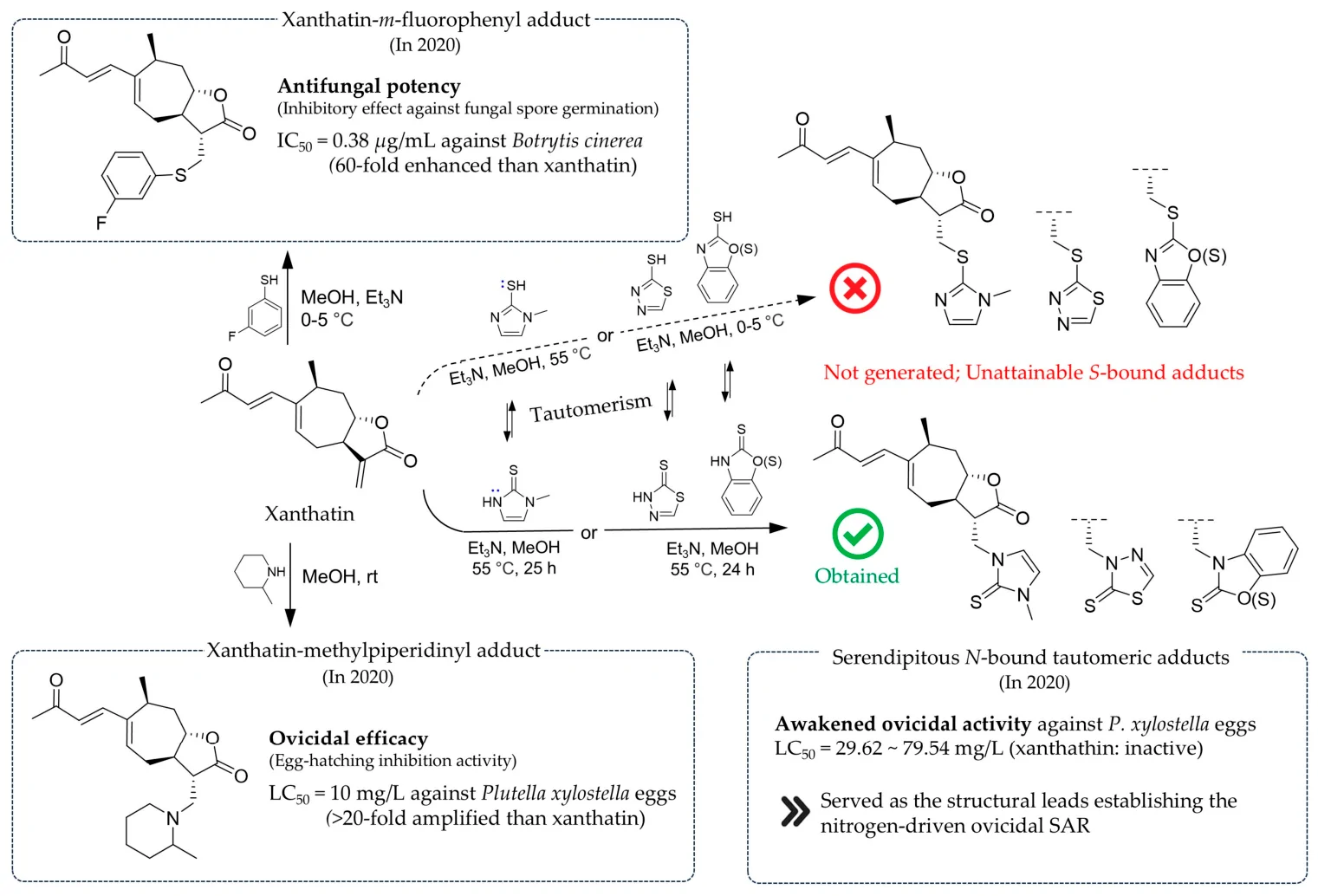
Divergent structural optimization of xanthatin via C-13 Michael addition: Tautomeric *N*-site expansion for enhanced ovicidal activity versus selective aromatic thiol optimization for potent antifungal activity.

Xanthatin is a prominent xanthanolide sesquiterpene lactone featuring a 5,7-fused bicyclic scaffold and a reactive α-methylene-γ-lactone warhead. As illustrated in Figure 15, Zhi et al. initially attempted to target the C-13 position via standard thiol-Michael addition to synthesize heterocyclic derivatives [20]. However, the reaction met a severe synthetic bottleneck, failing to generate the anticipated *S*-bound variants. Upon elevating the temperature to 55 °C, the dynamic equilibrium shifted toward an unexpected tautomeric modification, fully favoring heterocyclic *N*-site alkylation over the conventional *S*-attack. Although the intended *S*-bound adducts were unattainable, the resulting heterocyclic *N*-bound amino-Michael adducts successfully broke the biological dormancy of the parent core. While native xanthatin is completely inactive against *Plutella xylostella* eggs (LC_50_ > 200 mg/L), these serendipitous *N*-bound derivatives awakened robust ovicidal activity (LC_50_ = 29.62~79.54 mg/L), serving as the structural leads to establish the nitrogen-driven ovicidal SAR.

Driven by this conceptual breakthrough, a rational two-track optimization program was implemented. On the amino-diversification track, expanding the library into aliphatic cyclic secondary amines yielded a highly optimized methylpiperidinyl derivative, which achieved a >20-fold amplified ovicidal potency (LC_50_ = 10 mg/L). Concurrently, on the thiol-diversification track, optimizing simple aromatic thiols led to the discovery of a *meta*-fluorophenyl adduct. Powered by a strong fluorine bioisostere effect, this derivative demonstrated an extraordinary 60-fold enhancement in antifungal potency against *Botrytis cinerea* (IC_50_ = 0.38 μg/mL), significantly outperforming both native xanthatin (IC_50_ = 22.31 μg/mL) and commercial fungicides. In contrast, all modified adducts displayed compromised larvicidal activity against *P. xylostella larvae*, confirming that an unmasked exocyclic double bond is indispensable for larvicidal mechanisms. In parallel with these ovicidal evaluations, Michael-type amino derivatives of xanthatin were subsequently reported to display substantially enhanced antifungal potency against agricultural pathogens [43].

### 2.12. Dehydroleucodine Derivatives

**Figure 16:**
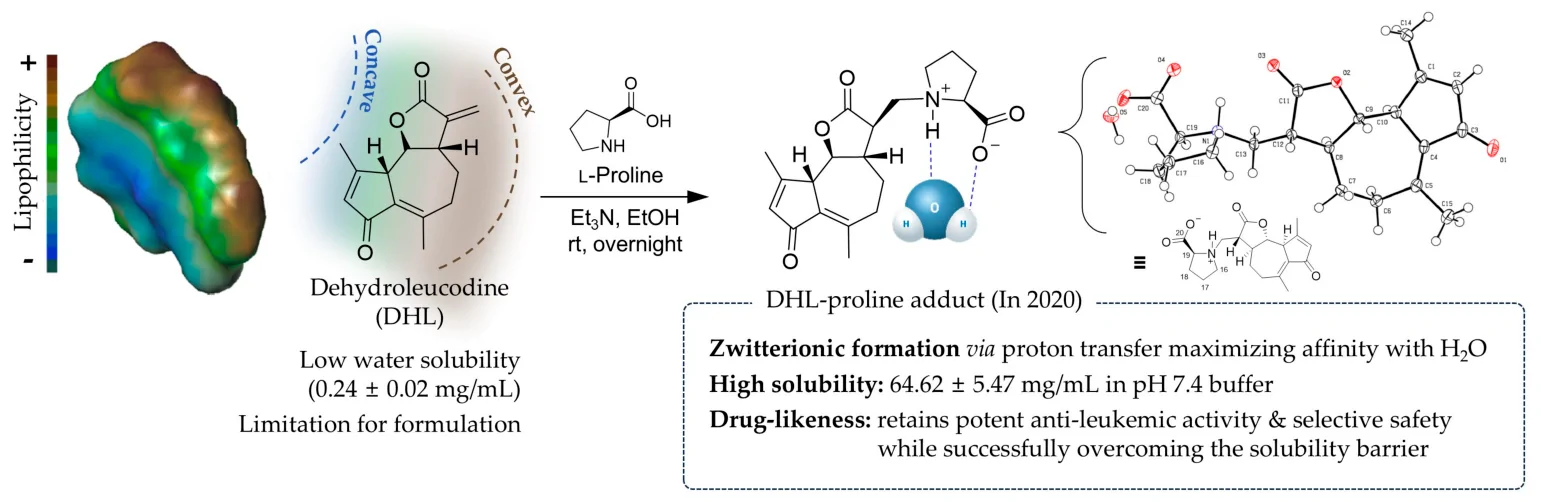
Schematic representation of the structural rationale for the enhanced water solubility of DHL-proline adduct.

Dehydroleucodine (DHL), a natural guaianolide sesquiterpene lactone, exhibits potent anti-leukemic activity, but its clinical translation has been hindered by extremely poor water solubility. Previous computational studies revealed that DHL features an asymmetric surface signature consisting of a convex lipophilic face and a concave hydrophilic face, which allows for cellular permeability but remains insufficient for absolute aqueous dissolution [44]. To overcome this biophysical limitation while maintaining therapeutic efficacy, an amino-prodrug strategy was employed to synthesize the DHL-proline adduct [21]. X-ray crystallographic and conformational analyses confirmed that this proline adduct undergoes an intramolecular proton transfer from the carboxyl group to the nitrogen atom, establishing a highly polar zwitterionic structure. This charge separation maximizes intermolecular ion-dipole and hydrogen-bonding interactions with H_2_O molecules, resulting in a dramatic ~270-fold increase in water solubility (64.62 ± 5.47 mg/mL) in pH 7.4 buffer. Strikingly, despite this profound modification of its lipophilic signature, the DHL-proline adduct fully retains its therapeutic potential, robustly downregulating NFκB transcription and upregulating oxidative stress biomarkers (HMOX1 and HSPA1A) to selectively eradicate acute myeloid leukemia cells while preserving normal mononuclear cell viability (Figure 16).

### 2.13. Arteannuin B Derivatives

To address the therapeutic limitations of native arteannuin B—a cadinane-type sesquiterpene lactone carrying an active α-methylene-γ-lactone warhead—a series of pharmacophoric amine conjugates consisting of substituted piperidine and piperazine derivatives was strategically synthesized in 2020 via a stereospecific 1,4-aza-Michael addition under mild, neutral conditions [22]. Highly proliferative cancer cells preferentially utilize anaerobic glycolysis to convert glucose into pyruvate and subsequently into lactate, even in the presence of oxygen (the Warburg effect). This accelerated glycolytic machinery causes a massive extrusion of hydrogen ions (H^+^) into the extracellular microenvironment, which can be dynamically monitored as the extracellular acidification rate (ECAR). Among the synthesized library, the arteannuin B-piperidine adduct (specifically the 3-ethoxycarbonylpiperidine conjugate) emerged as the most promising lead platform, exhibiting significantly amplified and robust in vitro tumor cytotoxicity compared to the parent arteannuin B, particularly achieving a remarkable 2.2-fold potency increase in RD sarcoma cells (IC_50_ = 11.27 ± 0.04 μM vs. 24.84 ± 0.05 μM for the parent compound).

**Figure 17:**
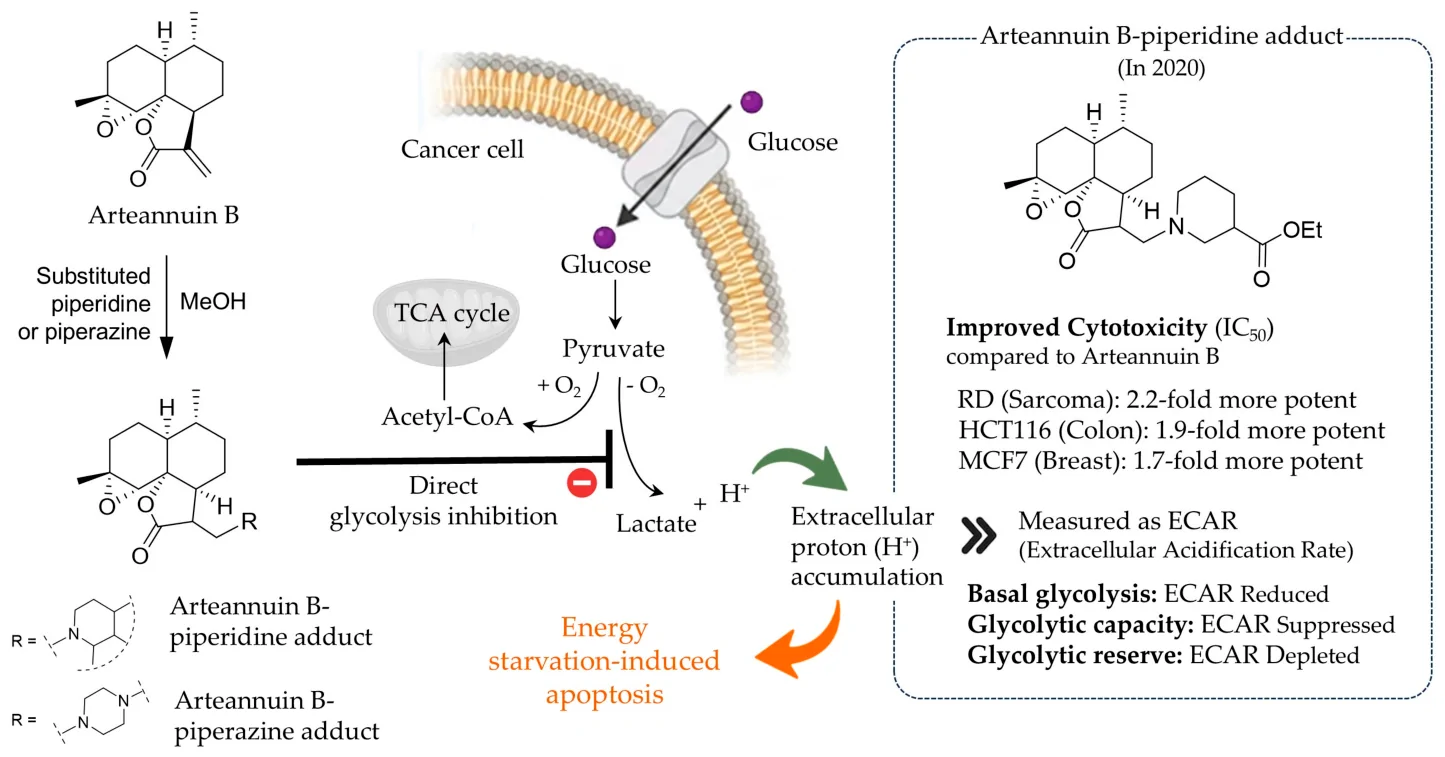
Proposed molecular mechanism of tumor glycolytic inhibition and metabolic blockade (ECAR suppression) by the lead arteannuin B-piperidine conjugate.

Mechanistically, real-time metabolic flux analysis using an Agilent Seahorse XFe96 analyzer revealed that this lead piperidine conjugate acts as a highly potent glycolytic inhibitor in MCF7 breast cancer cells. Unlike conventional SL adducts that typically act as reversible prodrugs, the prominent efficacy of this intact conjugate suggests its direct cytosol-specific intervention in the tumor’s metabolic machinery. Upon treatment, the arteannuin B-piperidine adduct successfully suffocated the glycolytic pathway by comprehensively reducing basal glycolysis, robustly suppressing glycolytic capacity, and completely depleting the glycolytic reserve, leading to a dramatic drop in the measured ECAR values. This multi-targeted metabolic disruption effectively deprives neoplastic cells of their primary anaerobic energy machinery, ultimately driving them toward energy starvation-induced tumor apoptosis (Figure 17).

### 2.14. Tomentosin Derivatives

**Figure 18:**
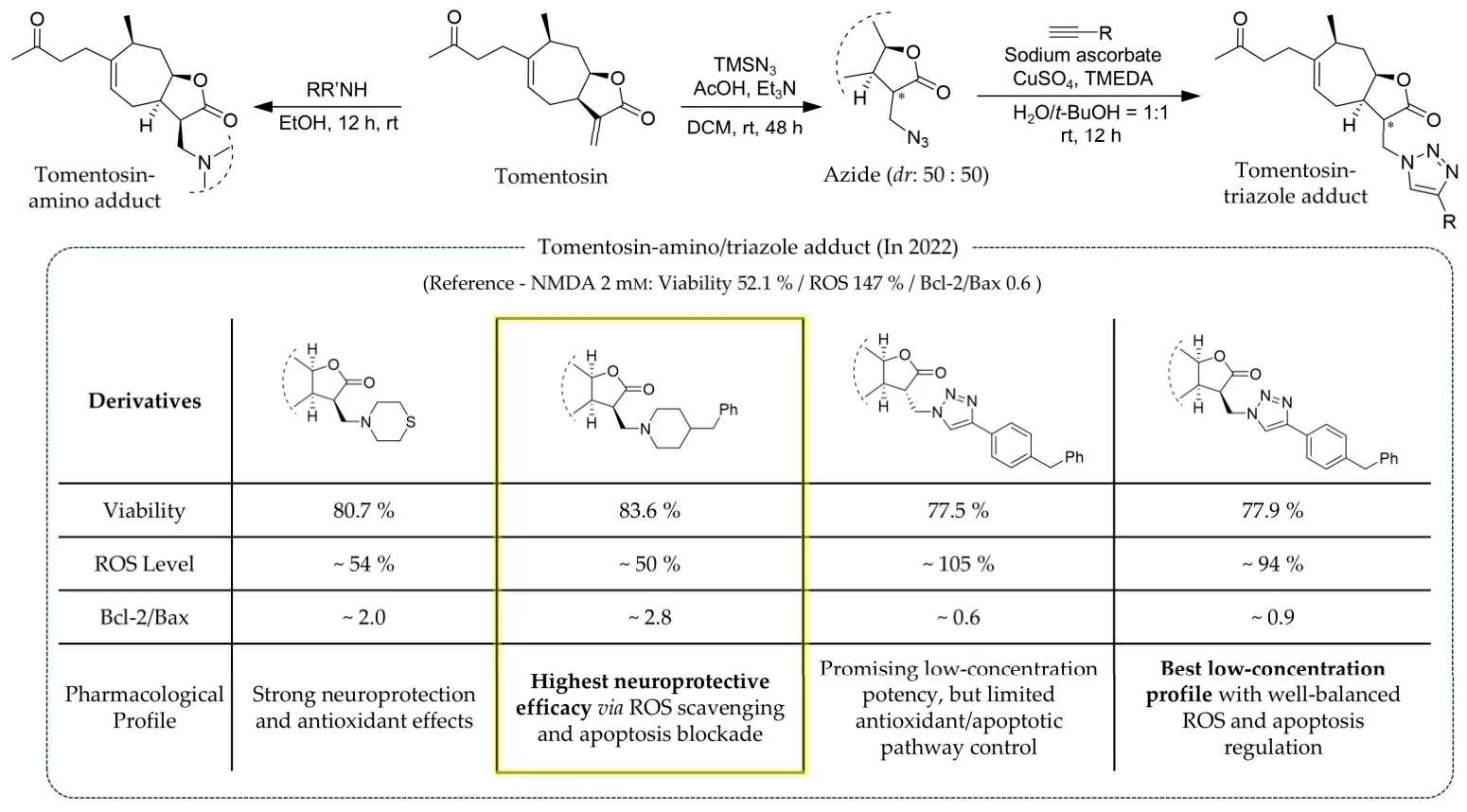
Synthetic pathways of amino- and 1,2,3-triazolo-tomentosin derivatives and their comparative pharmacological profiles.

Tomentosin, a sesquiterpene lactone extracted from *Dittrichia viscosa*, is a well-known natural scaffold with potent anti-inflammatory and cytotoxic properties. In 2022, this scaffold was strategically modified to suppress its inherent cytotoxicity while optimizing its protective efficacy against excitotoxicity [23]. While classical amino-conjugates were synthesized via well-established Michael addition reactions to serve as reversible prodrugs that gradually release the active parent structure in vivo, a key synthetic highlight is the introduction of a click chemistry-based approach. A Michael-type addition of TMSN_3_ to the α-methylene-γ-lactone system generated a 50:50 diastereomeric mixture of azide intermediates. The successful chromatographic separation of these diastereomers allowed for a precise structure–activity relationship investigation regarding the C11 stereocenters. As shown in Figure 18, while the amino-adducts generally required a higher concentration (1 μM) to exert robust cell viability restoration and ROS scavenging, the 1,2,3-triazole derivatives functioned as stable amide bioisosteres with high potency at a ten-fold lower concentration (0.1 μM). Notably, within the triazole series, one stereoisomer showed limited ROS and apoptotic pathway regulation despite preserving cell viability, whereas its separated counterpart exhibited an exceptional low-concentration profile by successfully restoring the Bcl-2/Bax ratio and suppressing ROS levels to near-basal states. This demonstrates that functional group masking, click scaffold incorporation, and stereoisomeric resolution are vital parameters for designing sophisticated multi-target neuroprotective agents. Providing a synthetic foundation for these neuroprotective evaluations, earlier studies successfully established 1,2,3-triazole-substituted tomentosins via copper-catalyzed click reactions on azide intermediates [45]. Building upon these promising anti-inflammatory and neuroprotective profiles, future investigations should further concentrate on advancing tomentosin derivatives, particularly by exploring their synergistic combinations with other therapeutic compounds and rigorously evaluating their targeted pharmacological efficacy across specific cell types.

## 3. Conclusions

The structural optimization of SLs via Michael addition has solidified its position as a highly successful paradigm in modern medicinal chemistry, offering a rational framework to overcome the long-standing biophysical and pharmacokinetic bottlenecks of these natural scaffolds. Characterized by an electrophilic α-methylene-γ-lactone core capable of covalent target interaction, native SLs possess privileged therapeutic potential; however, their clinical translation has been severely hindered by a narrow therapeutic window, poor solubility in aqueous media, and high systemic toxicity. Over the past decades, chemical functionalization strategies have systematically diverged into two major pathways. The first pathway focuses on constructing reversible amine or thiol adducts to mask the reactive warhead. This transient conjugate addition introduces a basic nitrogen or polar tail, which drastically improves solubility in aqueous media and oral bioavailability, serving as a classic prodrug platform that gradually regenerates the active parent structure in vivo through retro-Michael β-elimination. Among the various templates explored, PTL serves as the most extensively investigated archetype demonstrating the clinical viability of this approach. While native PTL suffers from negligible gastrointestinal absorption and remains undetectable in systemic circulation, its dimethylamine adduct (DMAPT) achieved a greater than 1000-fold increase in hydrophilicity and approximately 70% oral bioavailability. PTL optimization has further evolved toward highly sophisticated modifications, such as the tyramine adduct displaying single-digit nanomolar leukemia selectivity, and advanced pyrimidine-piperidine heterocycles yielding optimized Caco-2 permeability. Importantly, the nucleofugality of the transiently attached nucleophile, which directly correlates with the p*K*_a_ of its conjugate acid, dictates the balance between in vivo plasma stability and retro-Michael elimination rate. Choosing nucleophiles with a balanced p*K*_a_, such as cyclic secondary amines, provides adequate systemic half-life while enabling efficient thiol-triggered activation at target sites.

In this context, by addressing a clear literature gap that has remained unaddressed since earlier foundational reviews, the primary significance of this focus review lies in its systematic consolidation of these disparate synthetic strategies up to the first half of 2026, bridging the historical gap between classical organic chemistry and modern, target-specific drug design. By mapping the precise correlations between specific chemical modifications, such as stable 1,2,3-triazole amide bioisosteres or structurally rigid heterocyclic tails, and their resulting pharmacological outcomes, this work serves as an essential strategic roadmap for medicinal chemists. It moves the field beyond mere trial-and-error derivatization, providing a predictive framework to rationally tune the thermodynamic and kinetic parameters governing conjugate elimination. Ultimately, balancing reversible prodrug release with stable, structurally fixed pharmacophores, as illuminated through this comprehensive analysis, represents a highly sophisticated avenue to expand the narrow therapeutic windows of these privileged natural products, thereby accelerating the transition of SLs from promising natural leads into targeted, clinically viable therapeutics. Underscoring this growing therapeutic versatility, recent investigations up to 2024–2026 have expanded the scope of sesquiterpene lactone Michael adducts to include metabolic regulation, such as PKM2 activation for targeted anti-ulcerative colitis therapy [46].

## Figures and Tables

**Table 1:** Overview of sesquiterpene lactone derivatives synthesized via Michael addition and their pharmacological improvements.

Entity	Derivative	Parent Compound	Target Protein /Disease Model	Drug–Target Interactions/ Mechanisms	Quantitative Improvement	Ref. (Year)
1	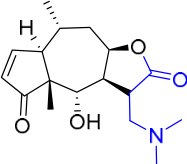	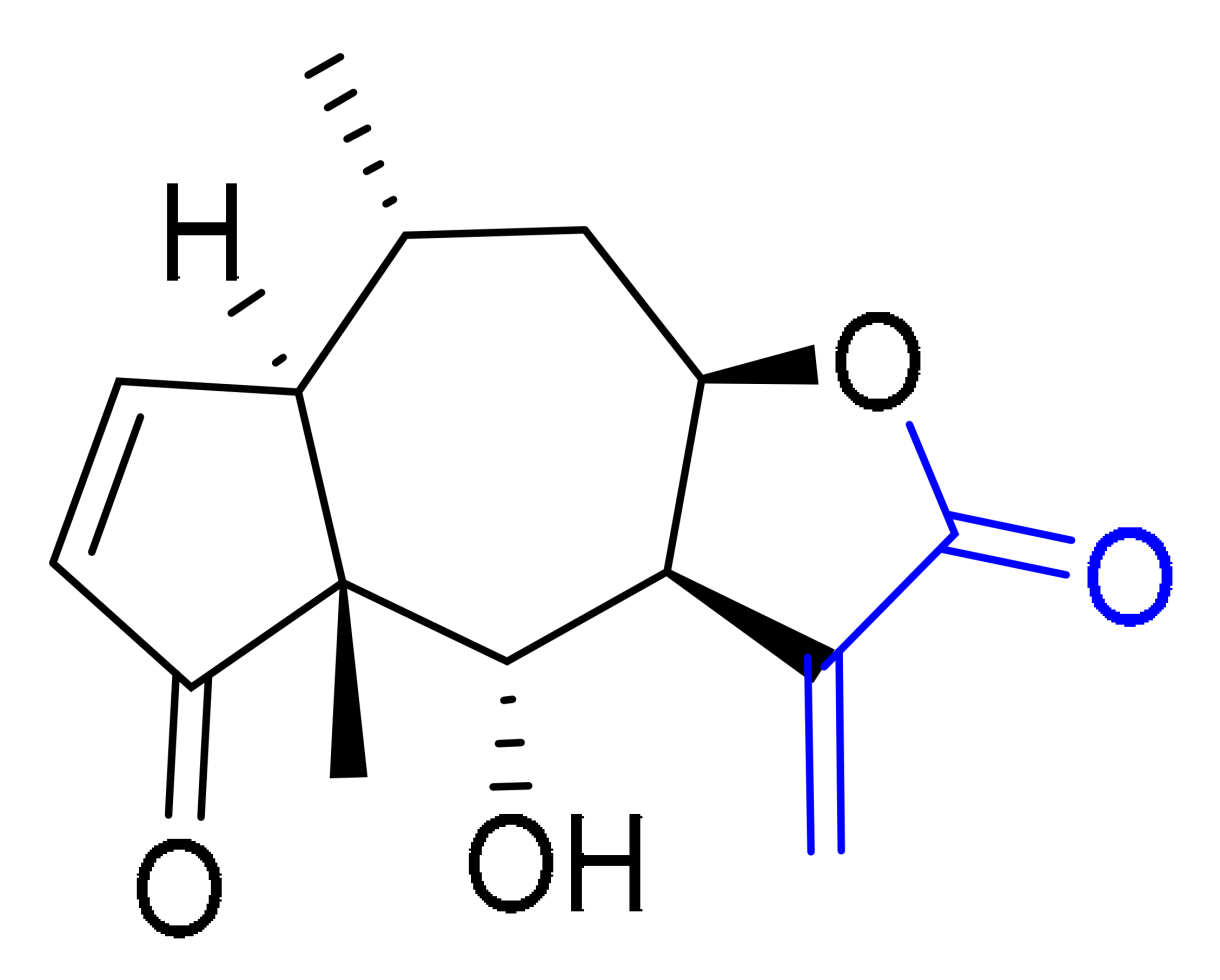	NF-κB/Leukemia & Larynx carcinoma	Alkylates tumor intracellular thiol pools to deplete G-SH, leading to ROS accumulation and covalent inhibition of NF-κB (p65 subunit, Cys38).	Maintained in vivo efficacy (mice survival: 13.0 days vs. 13.4 days) despite an 8-fold loss in in vitro potency (ED_50_: 0.604 μg/mL from 0.083 μg/mL), indicating compensated PK profiles	[10] (1972, 1975, 1978)
	Helenalin dimethylamine adduct	Helenalin				
2	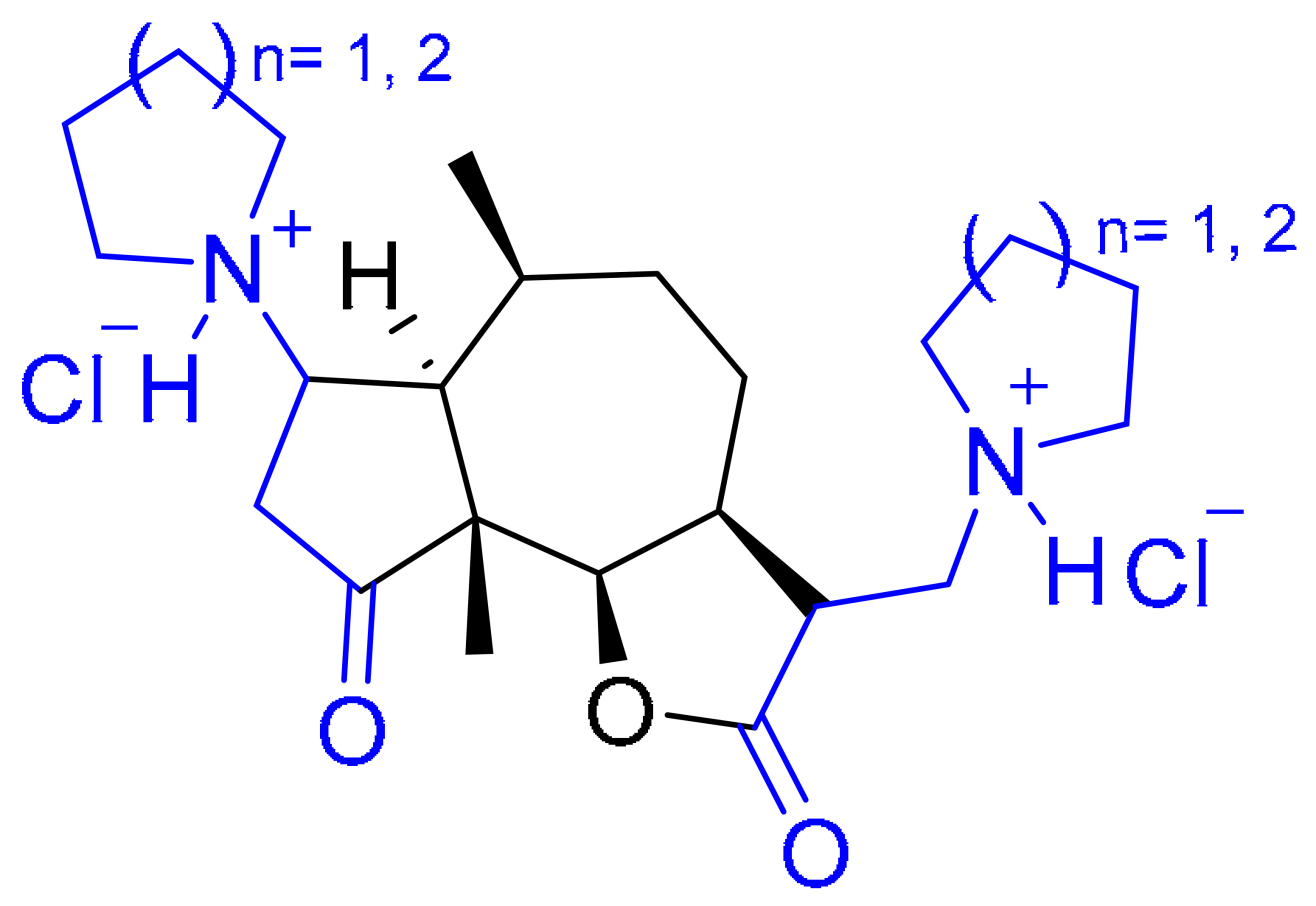	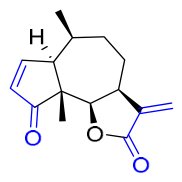	iNOS (Anti-inflammatory)/Cancer (e.g., Lung, Melanoma, Breast)	Post-Cope elimination, potently suppresses macrophage NO production via direct iNOS inhibition and direct NO radical scavenging	Substantially increased solubility in aqueous media; maintained comparable antiproliferative potency across 51 cancer cell lines	[11] (1995)
	Ambrosin bis-amine adducts	Ambrosin				
3	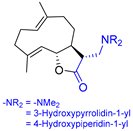	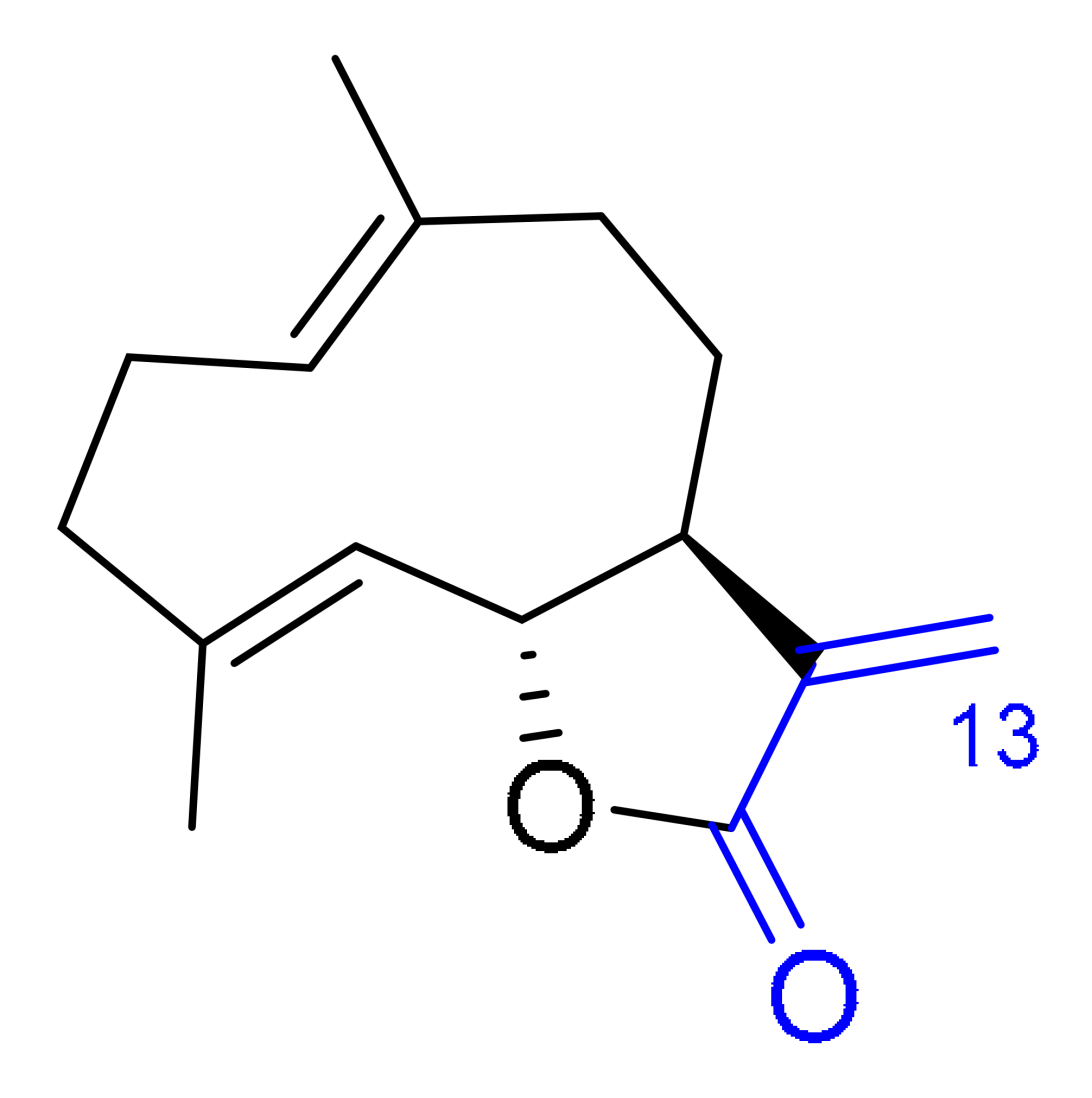	HSP72 (Phenotypic)/Cancer (e.g., Leukemia, Pancreatic, Ovarian)	React with target protein -SH groups to induce HSP72 expression, downregulating NF-κB activation; basic nitrogen tail enhances specific phenotypic cytotoxicity via hydrogen bonding	2- to 3-fold enhanced antiproliferative potency against sensitive cancer cells with an improved safety index (>2) toward normal cells.	[12] (2006)
	Costunolide amine adducts	Costunolide				
4	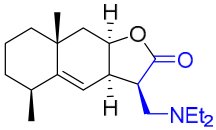	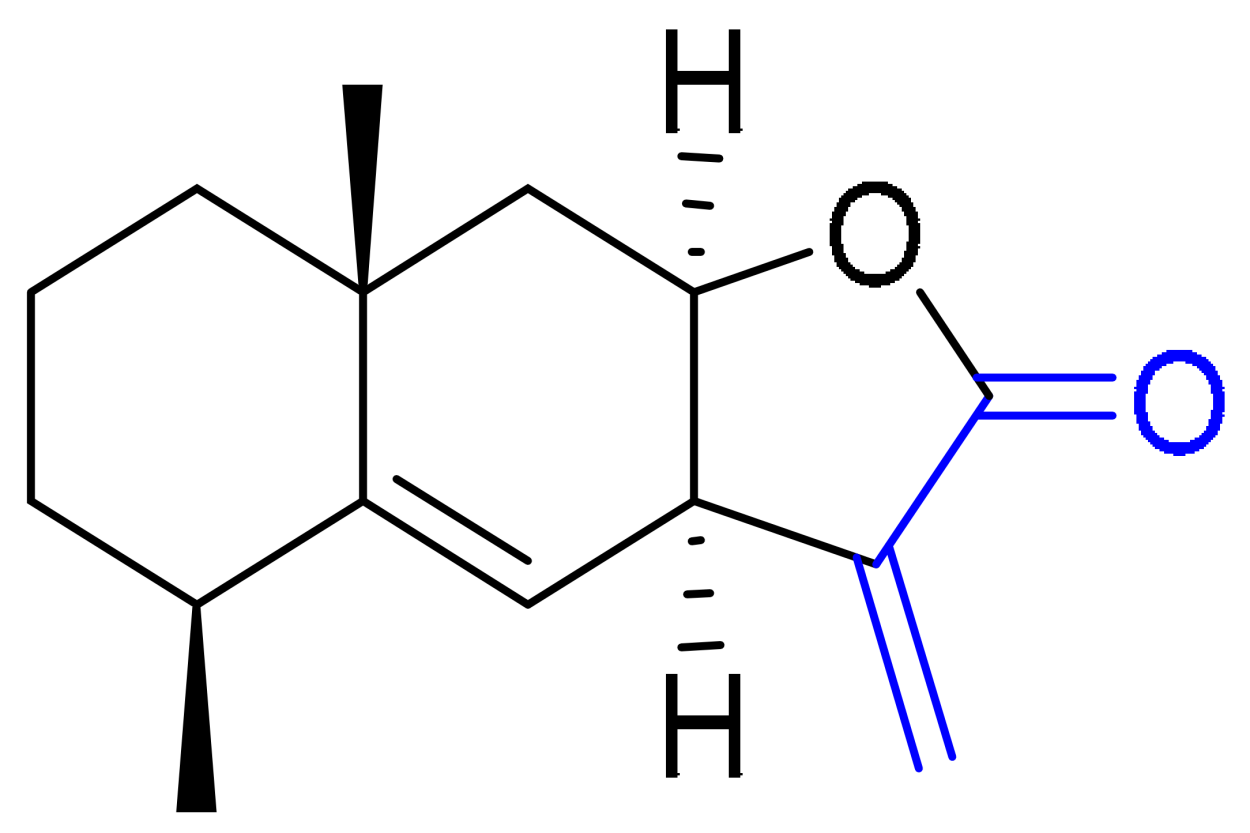	Unknown (Phenotypic; Alkylating agents)/Cancer (e.g., Leukemia, Breast, Lung)	Releases active parent cores to covalently cross-link with biological nucleophiles, triggering G2/M phase cell cycle arrest and apoptosis.	Maintained potent antiproliferative activities comparable to the parent compounds across multiple cell lines (e.g., K562 IC_50_: 0.7–2.0 μM) without significant loss of cytotoxicity	[13] (2001)
	Alantolactone diethylamine adduct	Alantolactone				
	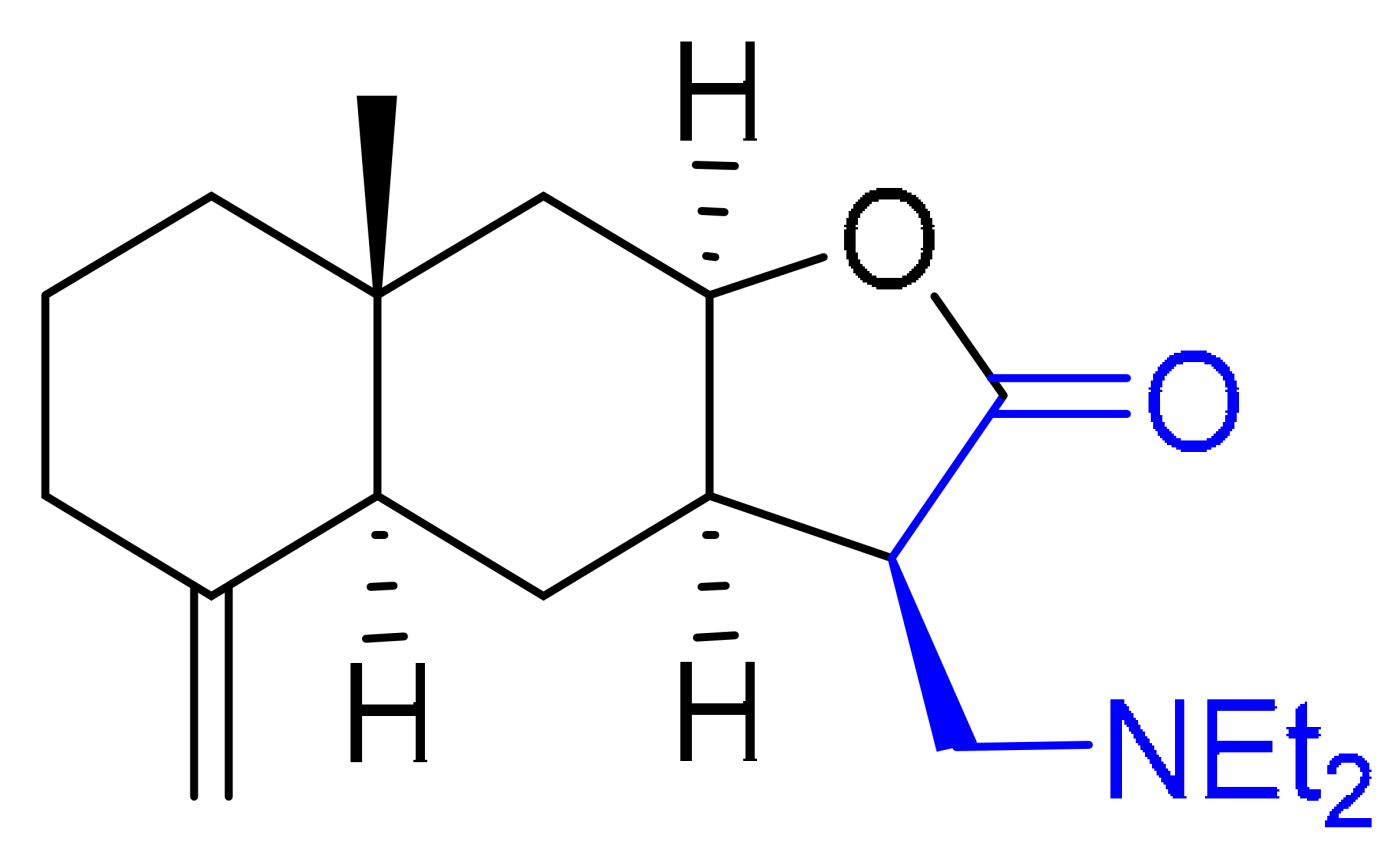	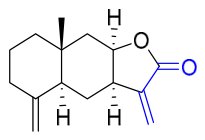				
	Isoalantolactone diethylamine adduct	Isoalantolactone				
5	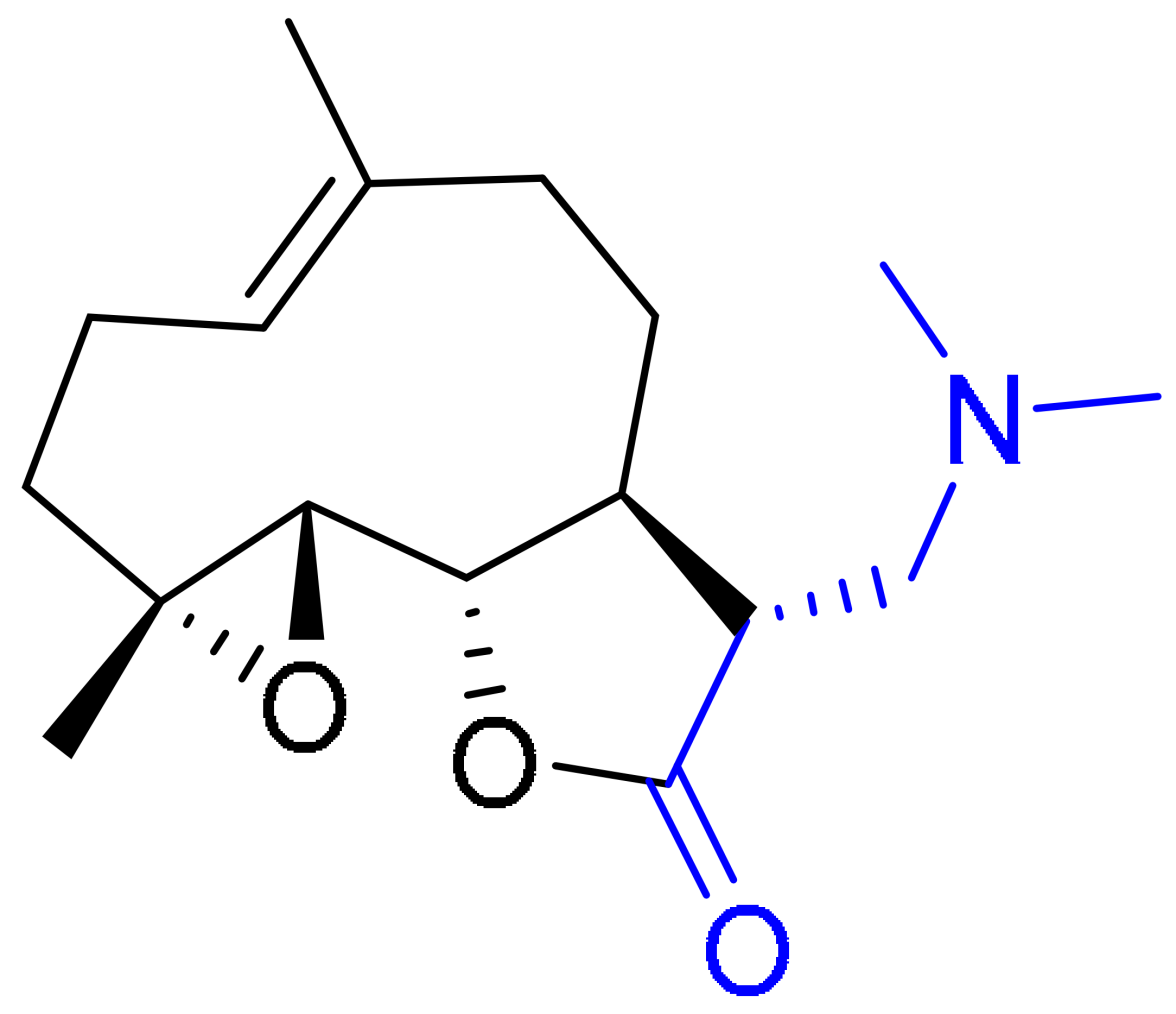	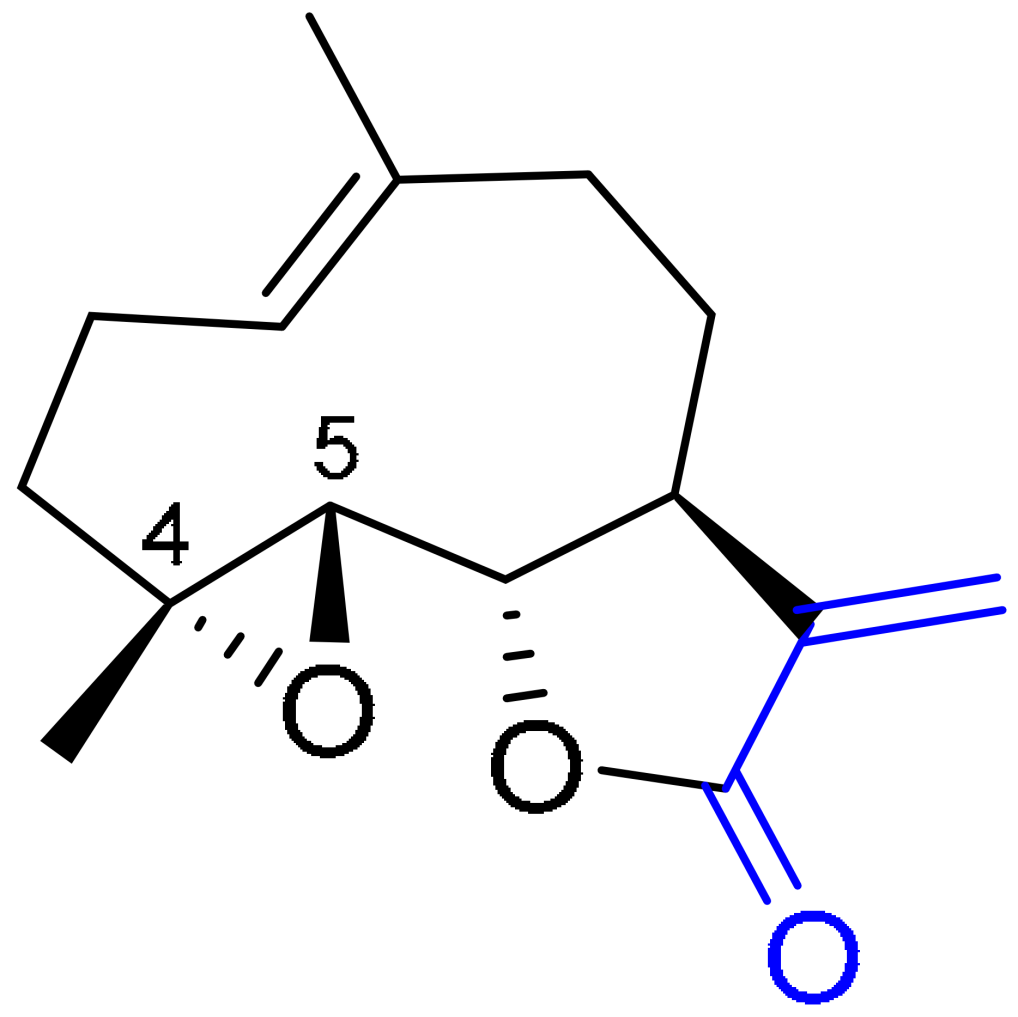	Unknown (Phenotypic)/Hepatitis C Virus (HCV) & Leukemia	Releases parent core in vivo to irreversibly alkylate nucleophilic proteomic residues (e.g., protein -SH groups)	>1000-fold increase in solubility in aqueous media and improved oral bioavailability; maintains potent antiviral/antiproliferative activity comparable to the parent compound	[14] (2006, 2007)
	Dimethylamino-parthenolide (DMAPT)	Parthenolide				
6	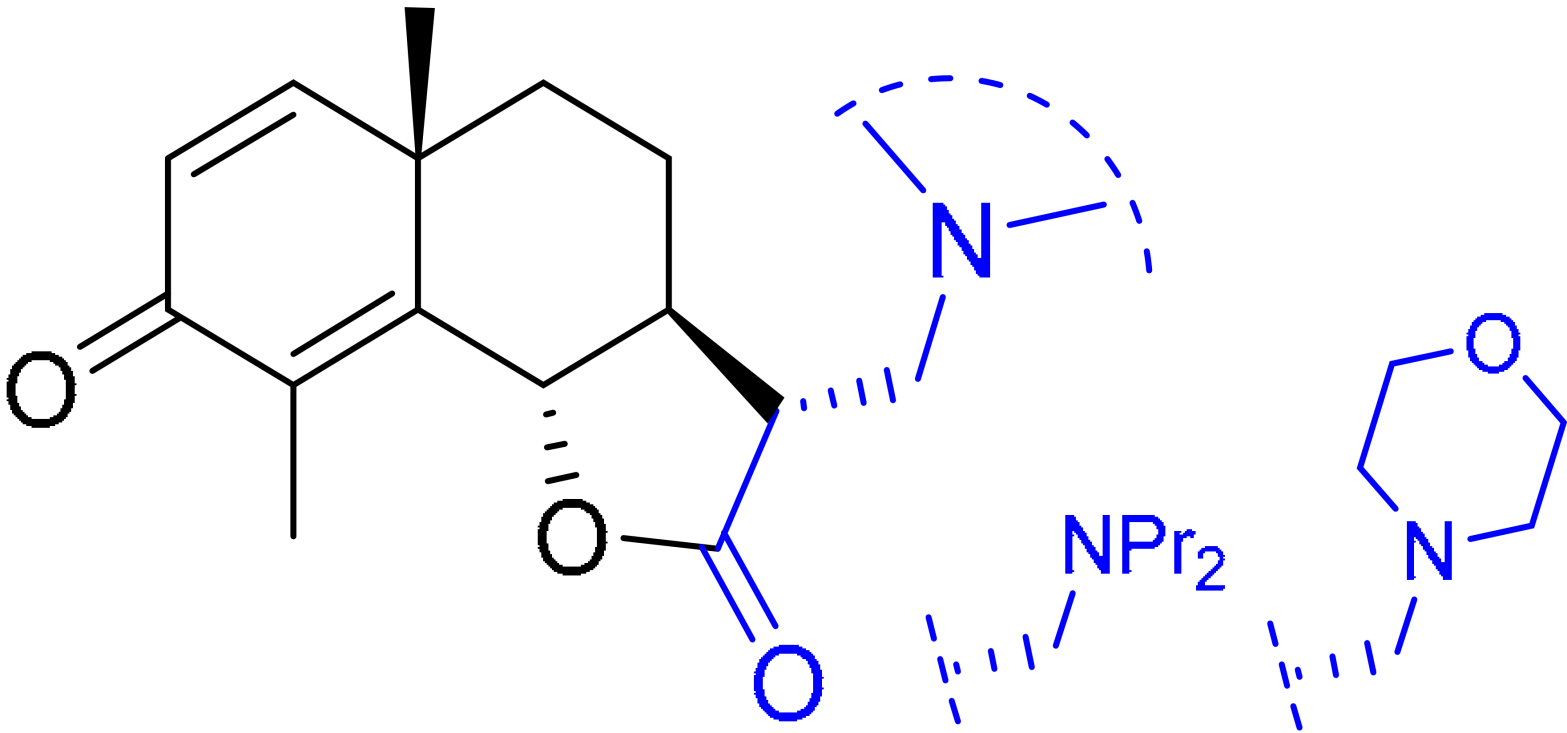	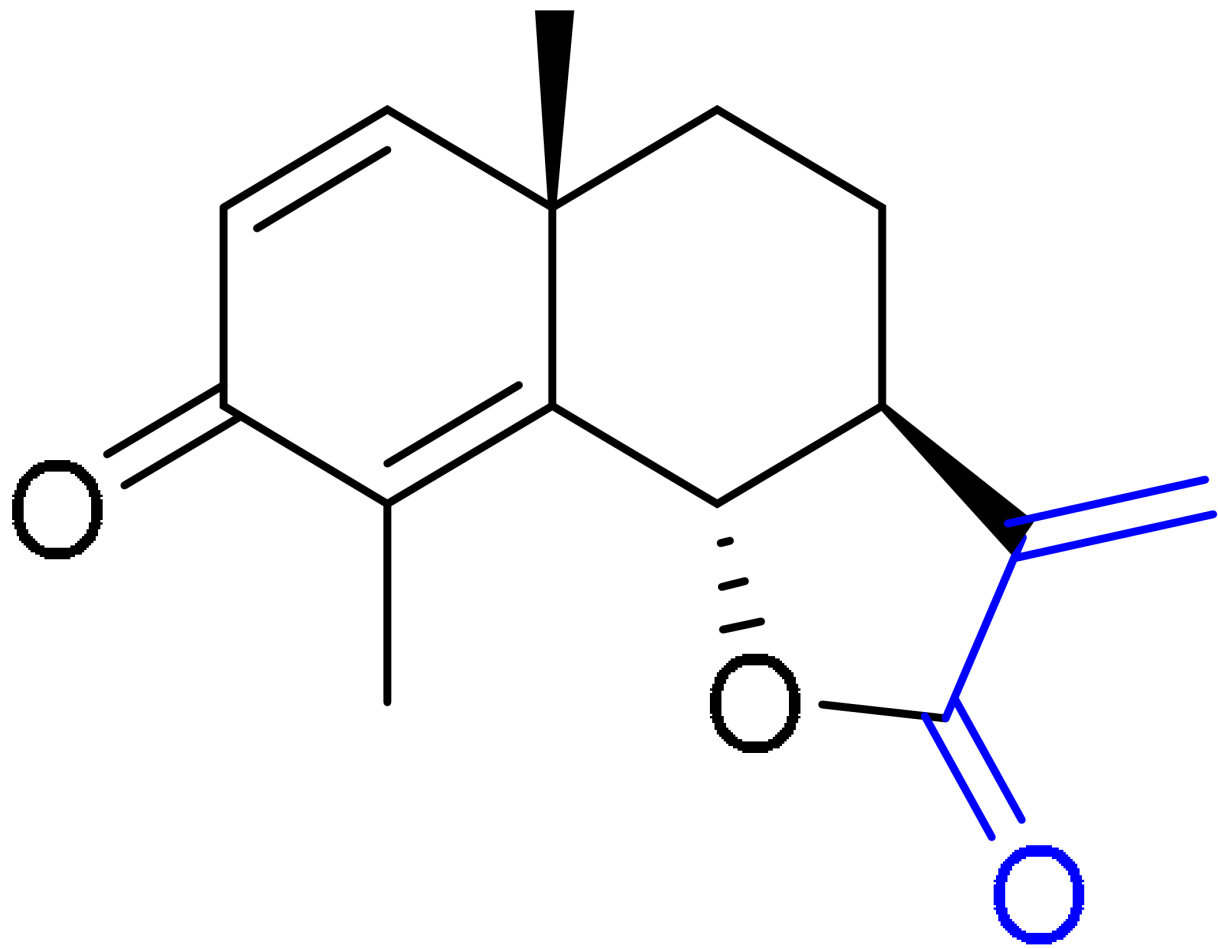	Unknown (Phenotypic)/Cancer (e.g., Human Melanoma MS, Ovary Adenocarcinoma CaOv)	Electrophilic warhead directs regioselective alkylation of biological nucleophiles (e.g., protein -SH groups) in phenotypic screening.	Moderate to potent cytotoxic activity; selected derivatives (e.g., morpholine adduct) exhibit significant inhibitory potency (GI_50_ = 3 × 10^−7^ M) surpassing the parent SL scaffold	[15] (2009)
	Dehydrosantonin amine adducts	Dehydrosantonin				
7	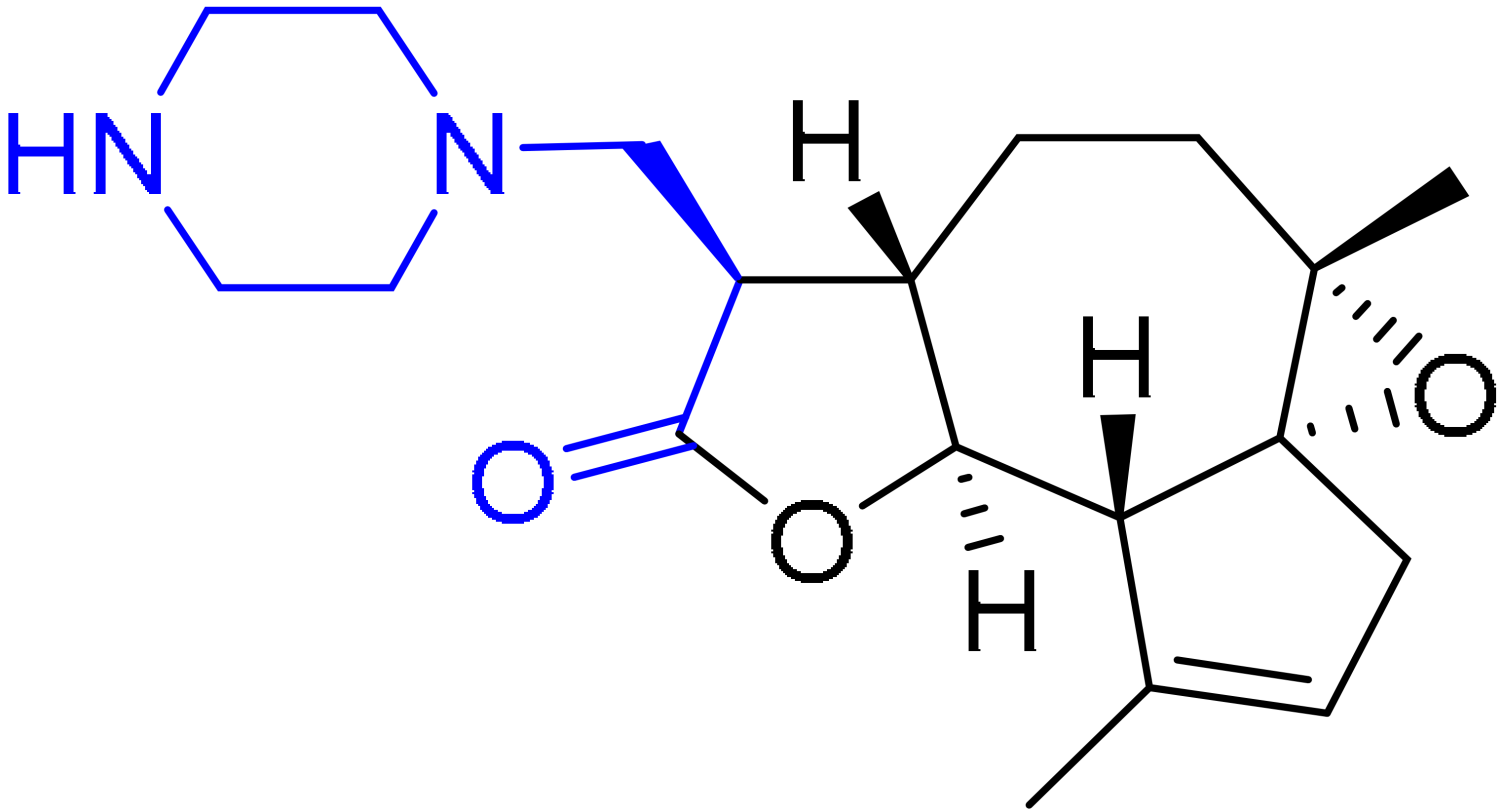	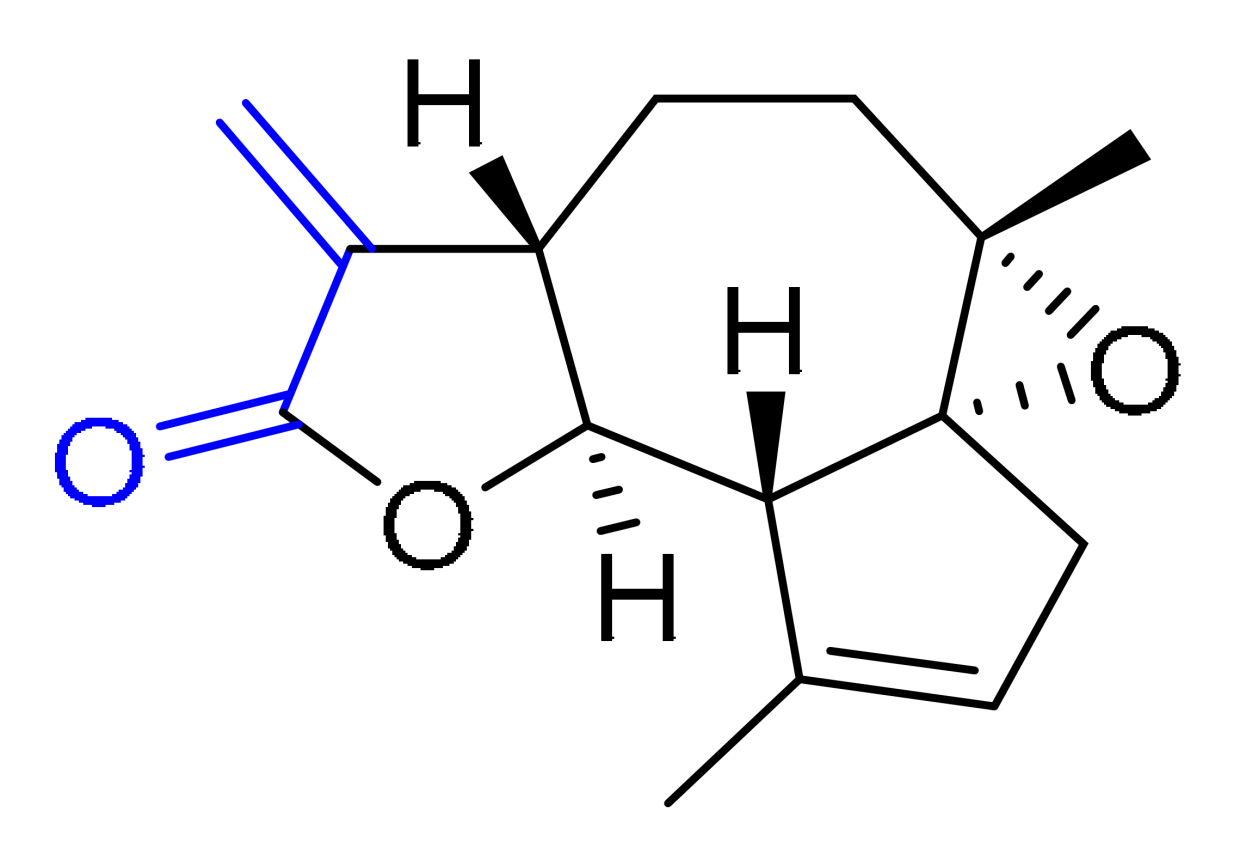	Ras pathway (farnesyltransferase inhibitor)/Cancer (e.g., Ovarian A2780, Lung A549)	Selectively blockades the Ras signaling pathway by inhibiting farnesyltransferase (FTase)- mediated post-translational modifications.	Potent cytotoxic activity; derivative shows enhanced selectivity (IC_50_ = 3.75 μM) compared to the parent scaffold (IC_50_ = 12.55 μM) in A2780 cells	[16] (2012)
	Arglabin piperazine adduct	Arglabin				
8	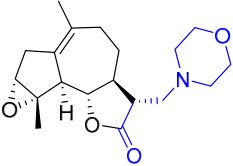	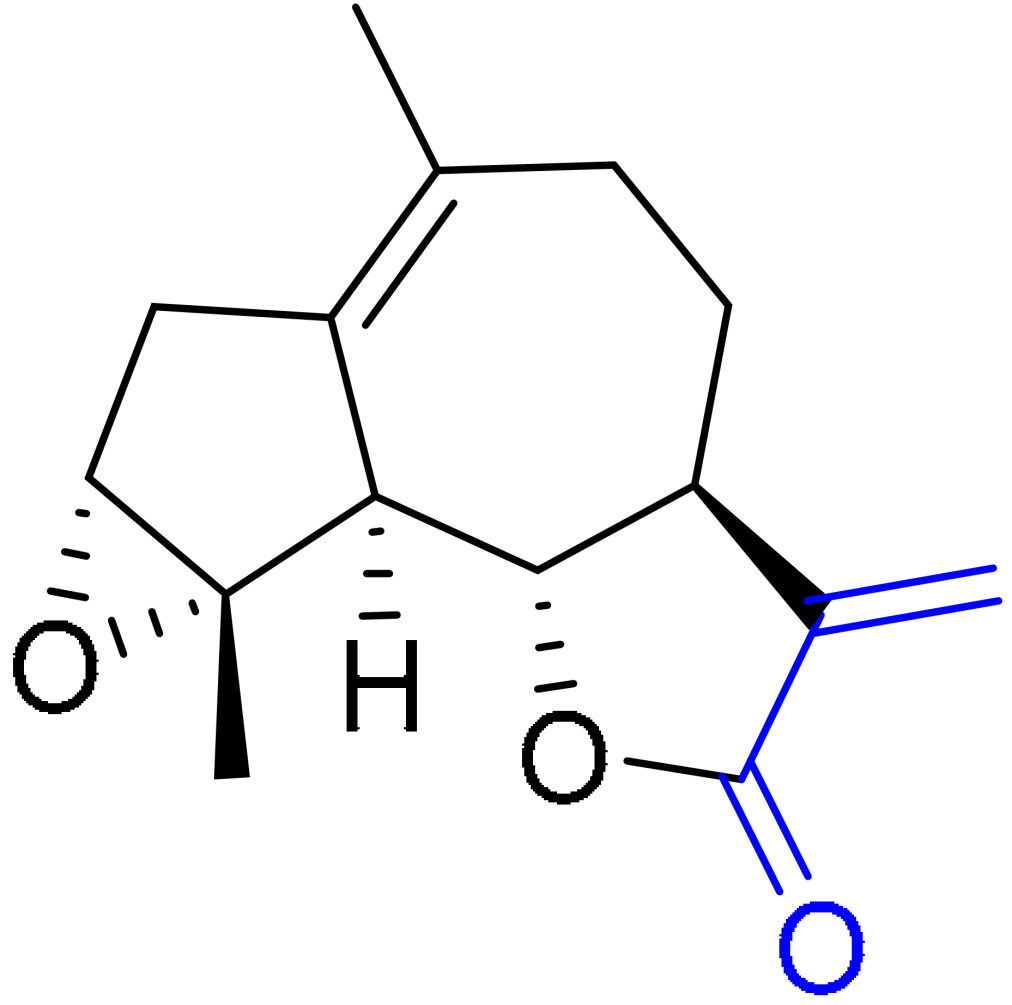	Ras pathway (farnesyltransferase inhibitor)/Leukemia (THP-1)	Actively inhibits downstream Ras protein farnesylation via competitive active-site occupancy, leading to leukemia cell death.	Potent and selective cytotoxic activity against Leukemia cells (IC_50_ = 2.8 μM)	[17] (2013)
	Ludartin morpholine adduct	Ludartin				
9	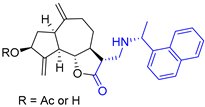	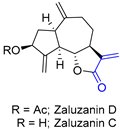	Intrinsic Apoptosis/Breast Cancer (MCF7, MDA-MB-231)	Intracellularly unmasks the active enone motif to trigger the mitochondrial-dependent intrinsic caspase-9/3 apoptotic cascade.	Naphthyl-based amino adducts provide enhanced antiproliferative potency compared to parent lactones; Zaluzanin C-adduct displays superior selectivity, sparing normal breast epithelial cells (MCF10A)	[18] (2018)
	Zaluzanin D/C-naphthyl adduct	Zaluzanin D/C				
10	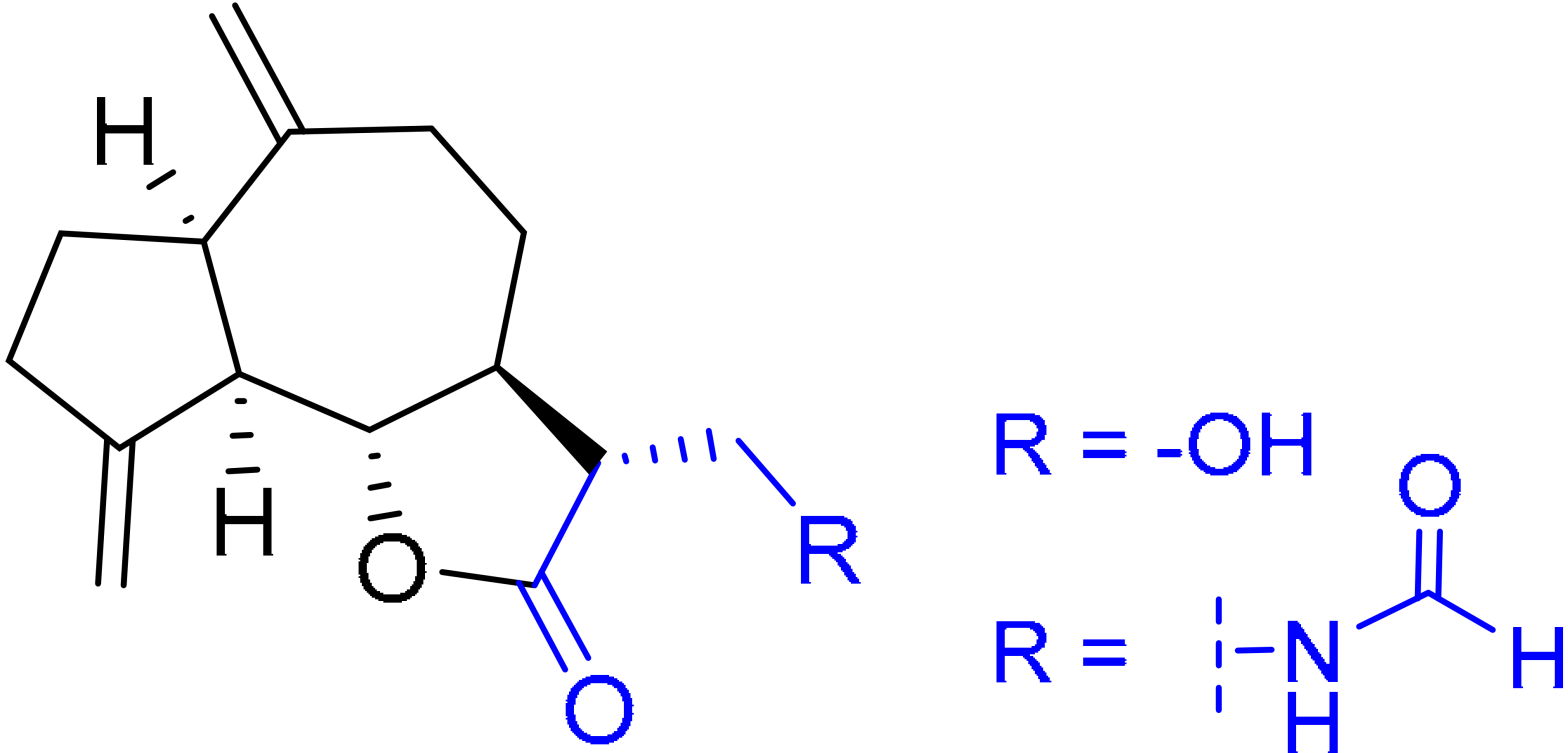	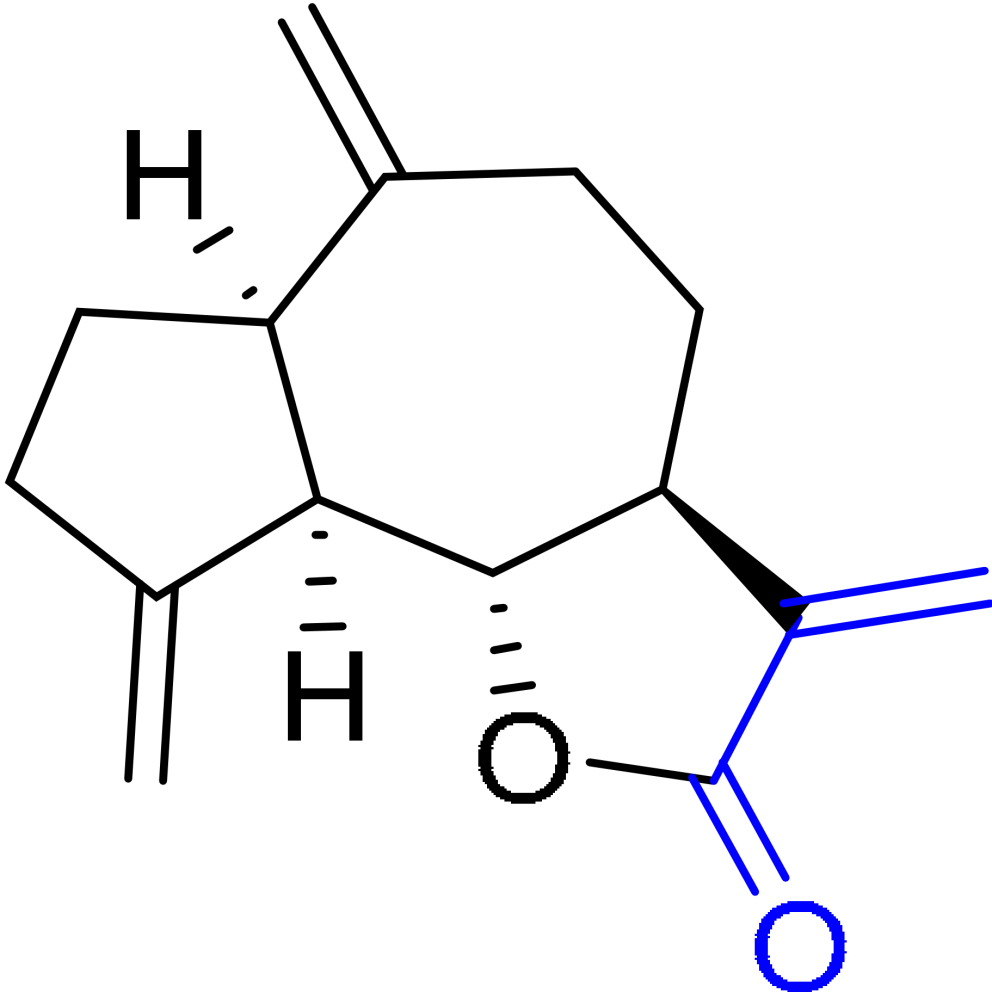	Parasitic weed seed germination (*P. ramosa* & *O. cumana*)	Skeleton-dependent electrophilic reactivity dictates selective stimulatory action via nucleophilic substitution at the core.	Guaiane-based derivatives show potent *P. ramosa* selectivity; Eudesmane-based derivatives exhibit superior stimulatory activity against *O. cumana*	[19] (2019)
	Hydroxy-/carbamoyl-dehydrocostuslactone	Dehydrocostuslactone				
	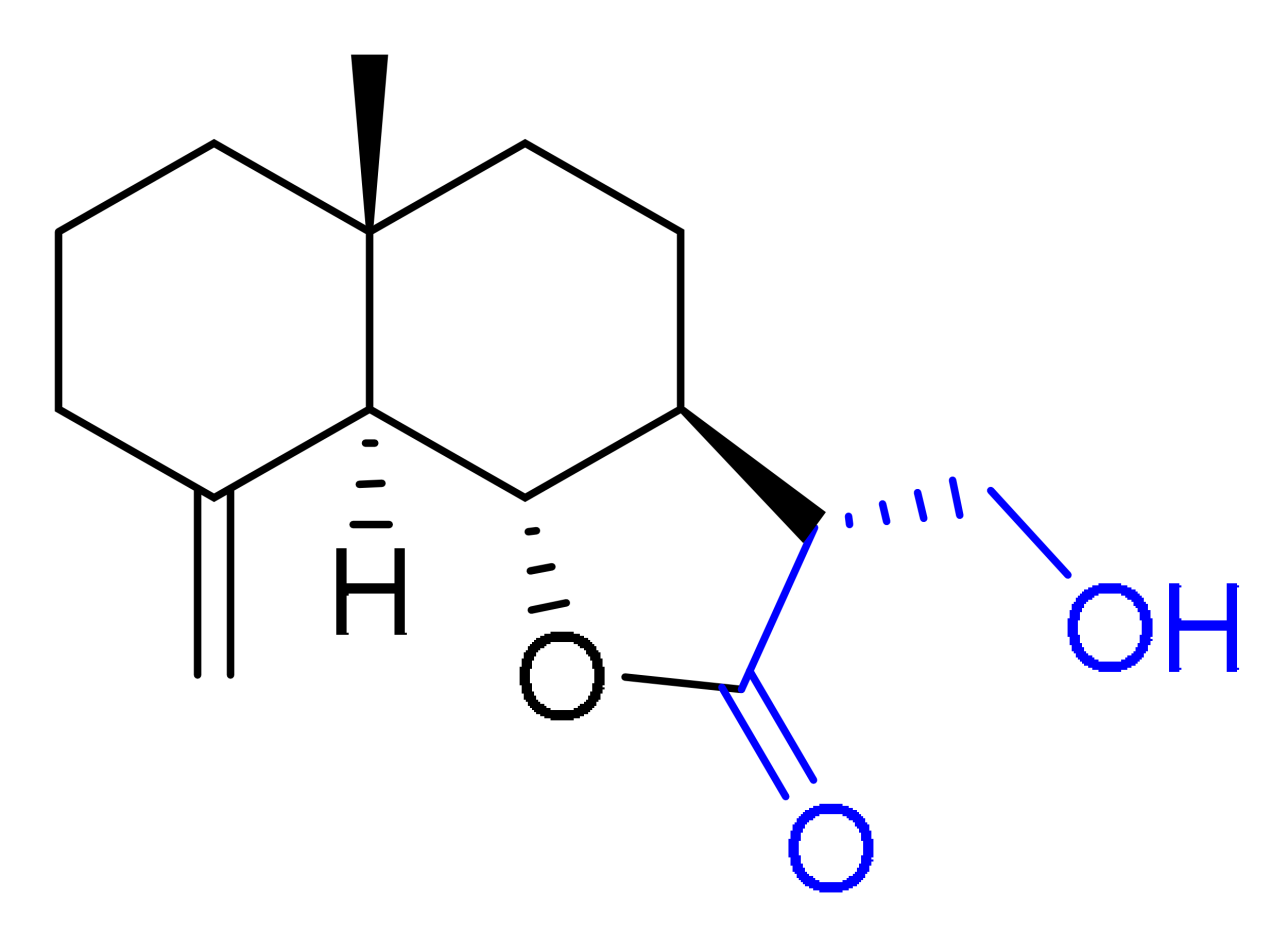	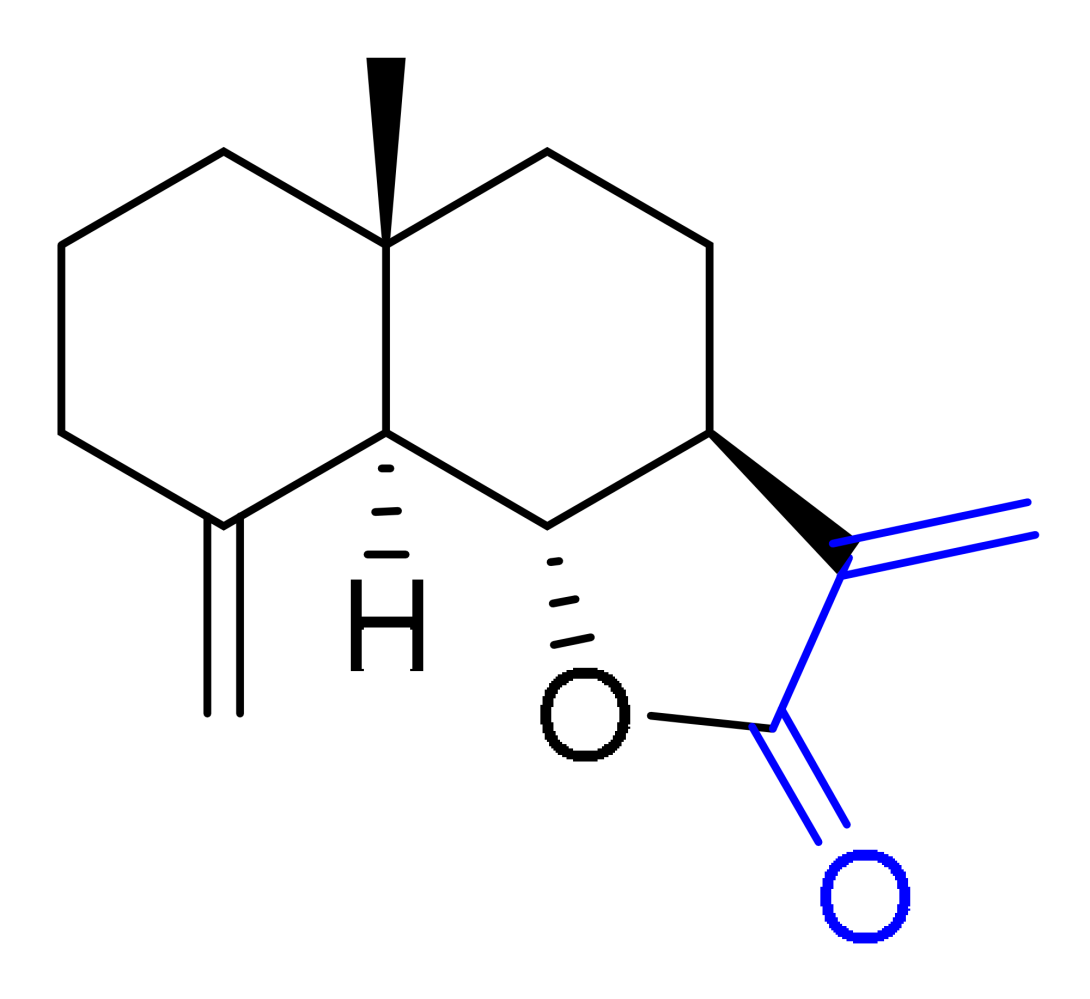				
	Hydroxy- β-Cyclocostunolide	β-Cyclocostunolide				
11	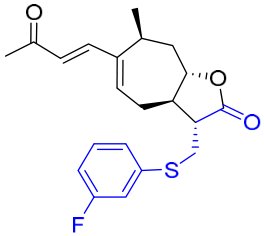	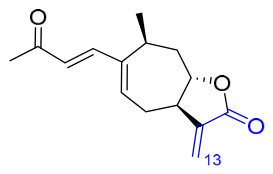	*Botrytis cinerea* (Gray mold)	Alters C-13 electrophilic reactivity to optimize targeted antifungal efficacy against agricultural pathogens.	Enhances antifungal potency (IC_50_ = 0.38 μg/mL), while significantly improving solubility in aqueous media for effective formulation in agricultural applications.	[20] (2020)
	Xanthatin-thio adduct	Xanthatin				
12	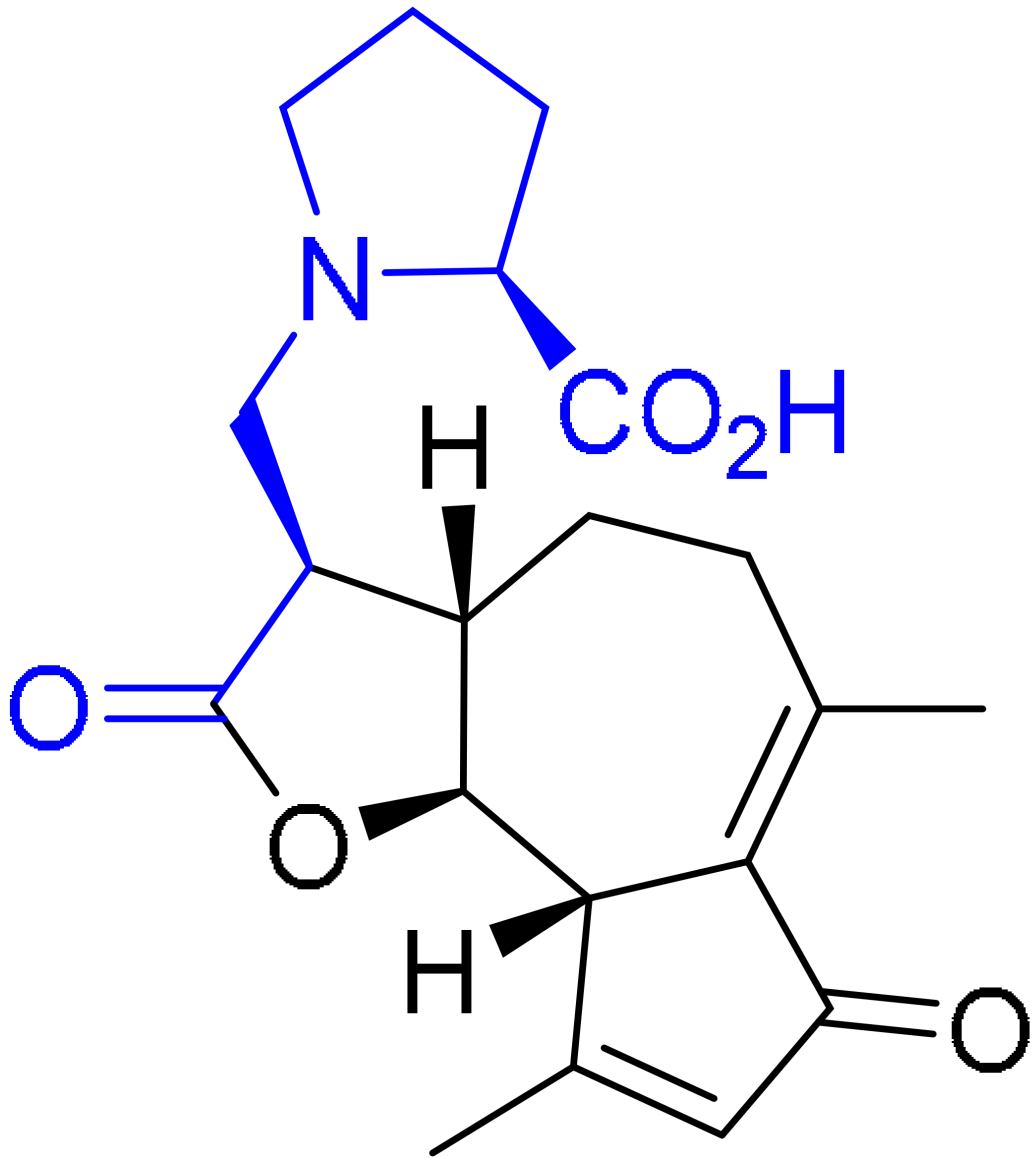	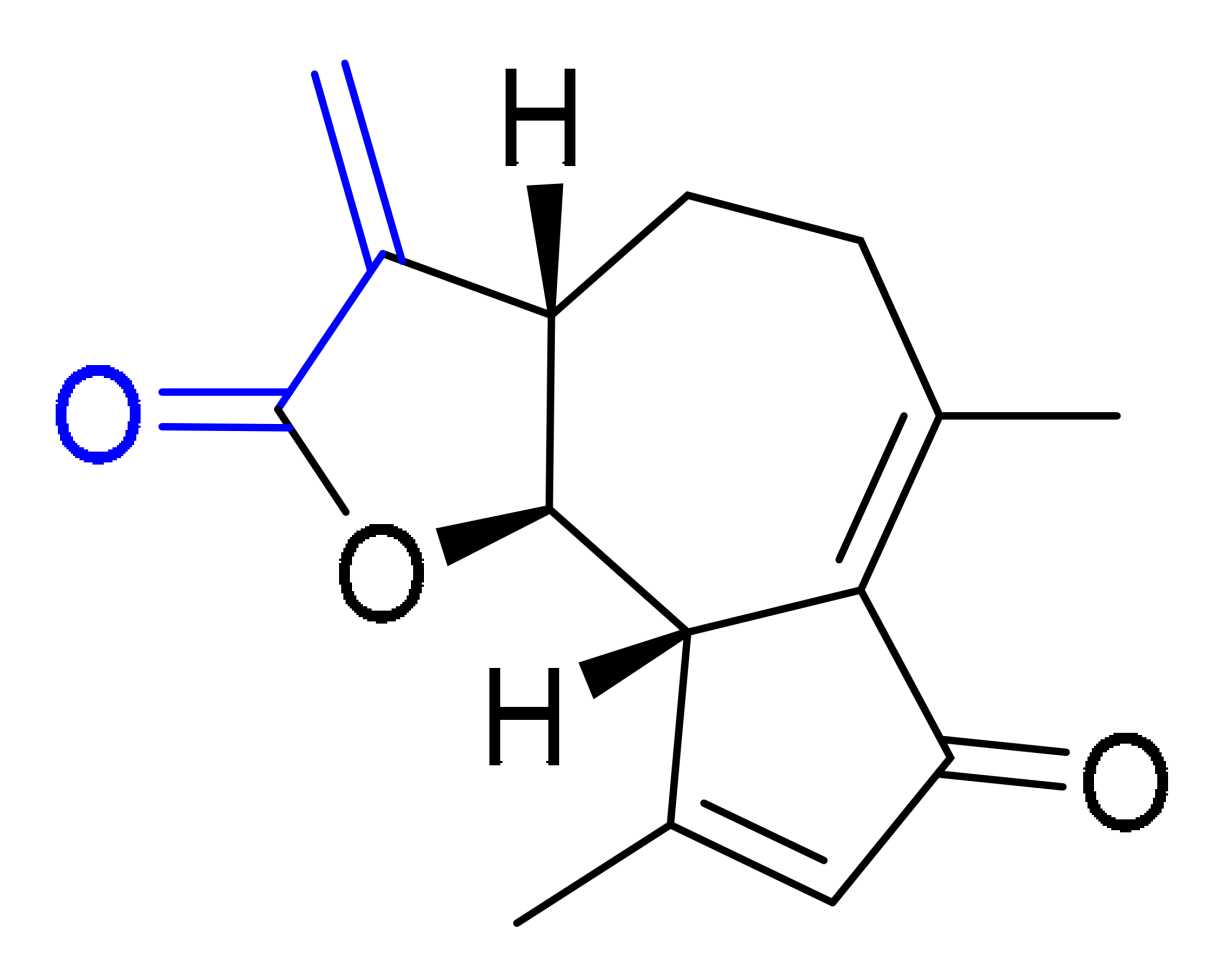	NF-κB/Acute Myeloid Leukemia (AML)	Covalently binds to NF-κB to trigger acute oxidative stress and robust upregulation of HMOX1 and HSPA1A.	Proline adduct demonstrates significant anti-leukemic activity and achieves an approx. 270-fold improvement in solubility in aqueous media	[21] (2020)
	DHL-proline adduct	Dehydroleucodine (DHL)				
13	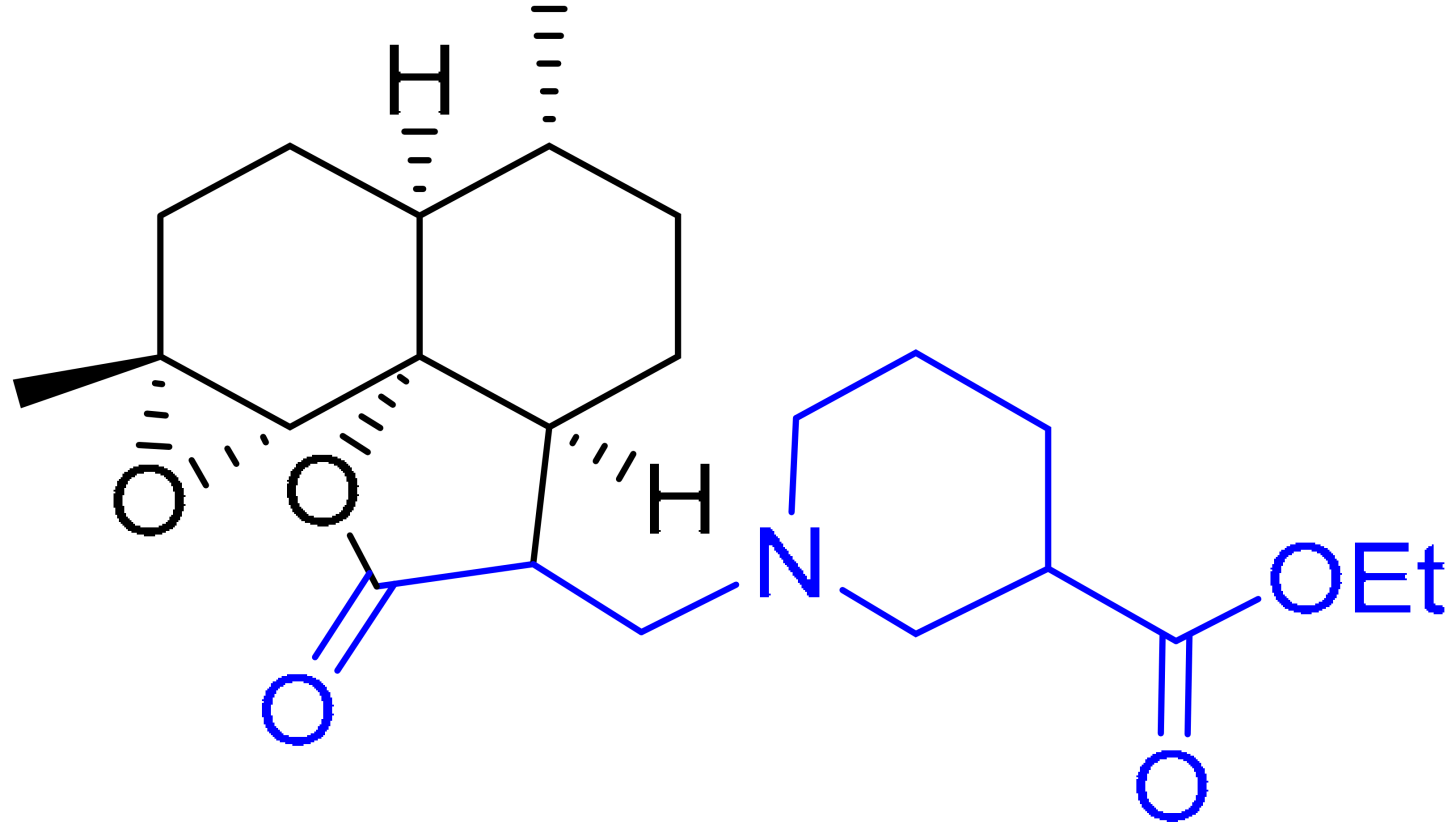	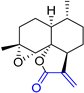	Glycolysis/Breast Cancer (MCF7)	Directly interferes with cancer cell metabolic machinery, comprehensively suffocating the anaerobic glycolysis pathway (ECAR depletion).	Identified as a lead compound with potent antiproliferative activity (IC_50_ = 8–36 μM; superior efficacy in modulating glycolytic capacity compared to parent compound	[22] (2020)
	Arteannuin B-piperidine adduct	Arteannuin B				
14	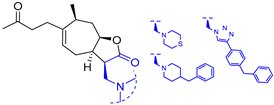	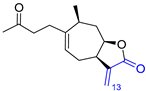	NMDA receptor/Excitotoxicity	Functions as a stable amide bioisostere that regulates the Bcl-2/Bax-mediated apoptotic cascade and mitigates NMDA receptor-induced oxidative stress.	Potent neuroprotective efficacy; compounds significantly restore neuronal cell viability and mitigate NMDA-induced damage	[23] (2022)
	Tomentosin-amino/triazole adduct	Tomentosin				

## Data Availability

No new data were created or analyzed in this study. Data sharing is not applicable to this article.
